# The effects of climate change on the Pleistocene rock art of Sulawesi

**DOI:** 10.1038/s41598-021-87923-3

**Published:** 2021-05-13

**Authors:** J. Huntley, M. Aubert, A. A. Oktaviana, R. Lebe, B. Hakim, B. Burhan, L. Muhammad Aksa, I. Made Geria, M. Ramli, L. Siagian, H. E. A. Brand, A. Brumm

**Affiliations:** 1 Griffith Centre for Social and Cultural Research, PERAHU, Griffith University, Gold Coast, QLD Australia; 2 Australian Research Centre for Human Evolution, Environmental Futures Research Institute, Griffith University, Brisbane, QLD Australia; 3 Pusat Penelitian Arkeologi Nasional (ARKENAS), Jakarta, Indonesia; 4 Balai Pelestarian Cagar Budaya, Sulawesi Selatan, Makassar, Indonesia; 5 Balai Arkeologi Sulawesi, Sulawesi Selatan, Makassar, Indonesia; 6 Museum Kepresidenan Republik Indonesia, Balai Kirti, Bogor, Indonesia; 7 Universitas Gadjah Mada (Fakultas Ilmu Budaya-Magister Arkeologi), Yogyakarta, Indonesia; 8 Australian Synchrotron, Clayton, VIC Australia

**Keywords:** Climate change, Palaeoclimate, Climate-change impacts, Archaeology, Climate sciences, Environmental sciences, Environmental social sciences, Materials science

## Abstract

The equatorial tropics house some of the earliest rock art yet known, and it is weathering at an alarming rate. Here we present evidence for haloclasty (salt crystallisation) from Pleistocene-aged rock art panels at 11 sites in the Maros-Pangkep limestone karsts of southern Sulawesi. We show how quickly rock art panels have degraded in recent decades, contending that climate-catalysed salt efflorescence is responsible for increasing exfoliation of the limestone cave surfaces that house the ~ 45 to 20-thousand-year-old paintings. These artworks are located in the world’s most atmospherically dynamic region, the Australasian monsoon domain. The rising frequency and severity of El Niño-induced droughts from anthropogenic climate change (that is, higher ambient temperatures and more consecutive dry days), combined with seasonal moisture injected via monsoonal rains retained as standing water in the rice fields and aquaculture ponds of the region, increasingly provide ideal conditions for evaporation and haloclasty, accelerating rock art deterioration.

## Introduction

The effects of human-induced climate change are increasingly expressed through environmental disasters across the globe^1,2^. While human health and security^2–4^ have rightly been research focuses, climate change equally impacts the long-term survival of physical remnants from the human past, our cultural heritage^5–7^. Here we argue that climate fluctuations over recent millennia and especially in recent decades, have been, and are increasingly, a major catalyst for the deterioration of Pleistocene cave art in Maros-Pangkep, a limestone karst area on the Indonesian island of Sulawesi. Uranium-series dating of rock art found in the caves and rock shelters of these near-coastal lowland karsts has demonstrated it is some of the earliest yet known anywhere on the planet, dating to a minimum 45.5 ka and including hand stencils and figurative depictions of animals, human/animal composites (therianthropes), as well as possibly the earliest known narrative scene in prehistoric art^8–11^. With more than 300 cave/shelter sites in the region known to preserve this Pleistocene style parietal art^8,11^, it is clear that the Maros-Pangkep assemblage rivals the celebrated ‘ice age’ cave art of western Europe, where scholars had until recently thought the oldest cave art traditions first emerged. We contend that climate-catalysed salt efflorescence (i.e., haloclasty or salt crystallisation) is responsible for the exfoliation of the older, case-hardened limestone surfaces of Maros-Pangkep’s cave/shelter sites, a process that is widespread throughout older karst cave surfaces in the broader Indonesian region^12–15^. Moreover, the exfoliation process, which destroys the rock surfaces or “canvases” on which the Late Pleistocene art was created, appears to have worsened in Maros-Pangkep in recent decades—a trend we believe is set to accelerate with warming ambient temperatures and increasingly frequent/severe El Niño events^16^. The extent of salt efflorescence in the 11 Maros-Pangkep sites we investigated, coupled with conservative forecasts for a 1.5 to 2 °C raise in global temperatures and accompanying extreme weather events^17,18^, have grave implications for the conservation of this globally significant cultural heritage. Aside from continuing limestone quarrying for the burgeoning domestic cement and *marmer* (marble) industries, global warming should be regarded as the greatest threat to the preservation of the ancient rock art that survives in Sulawesi and other parts of tropical Indonesia^8–11^.

**Figure 1:**
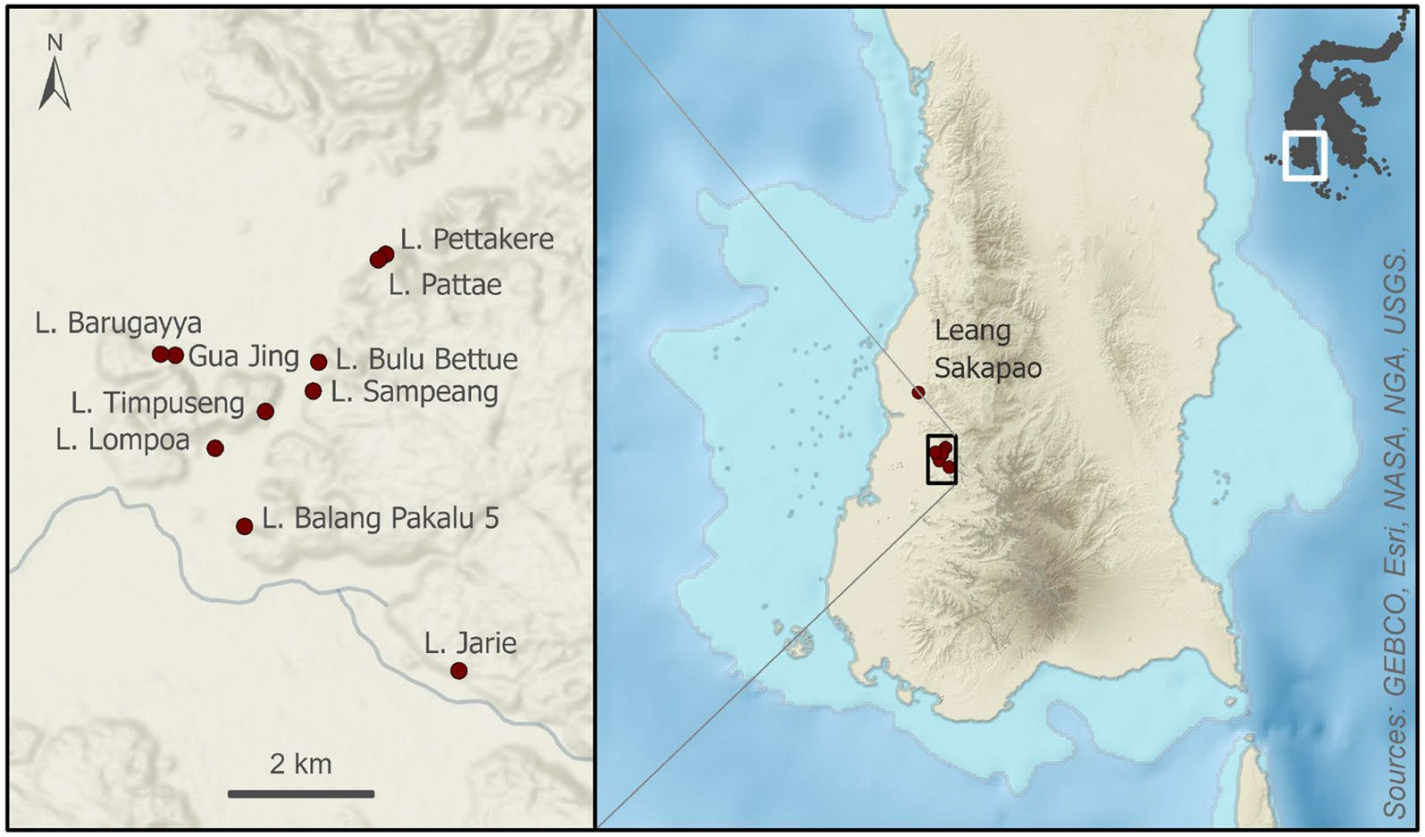
Location of the studied rock art sites in the limestone karsts of Maros-Pangkep, Sulawesi (Indonesia). The regional map (right) shows the insular shelf exposed during the Last Glacial Maximum. Inset map of Maros-Pangkep sites includes the location of a first/second order stream at the base of the karst. L is an abbreviation for Leang (Cave). Map created by Maria Kottermair.

**Figure 2:**
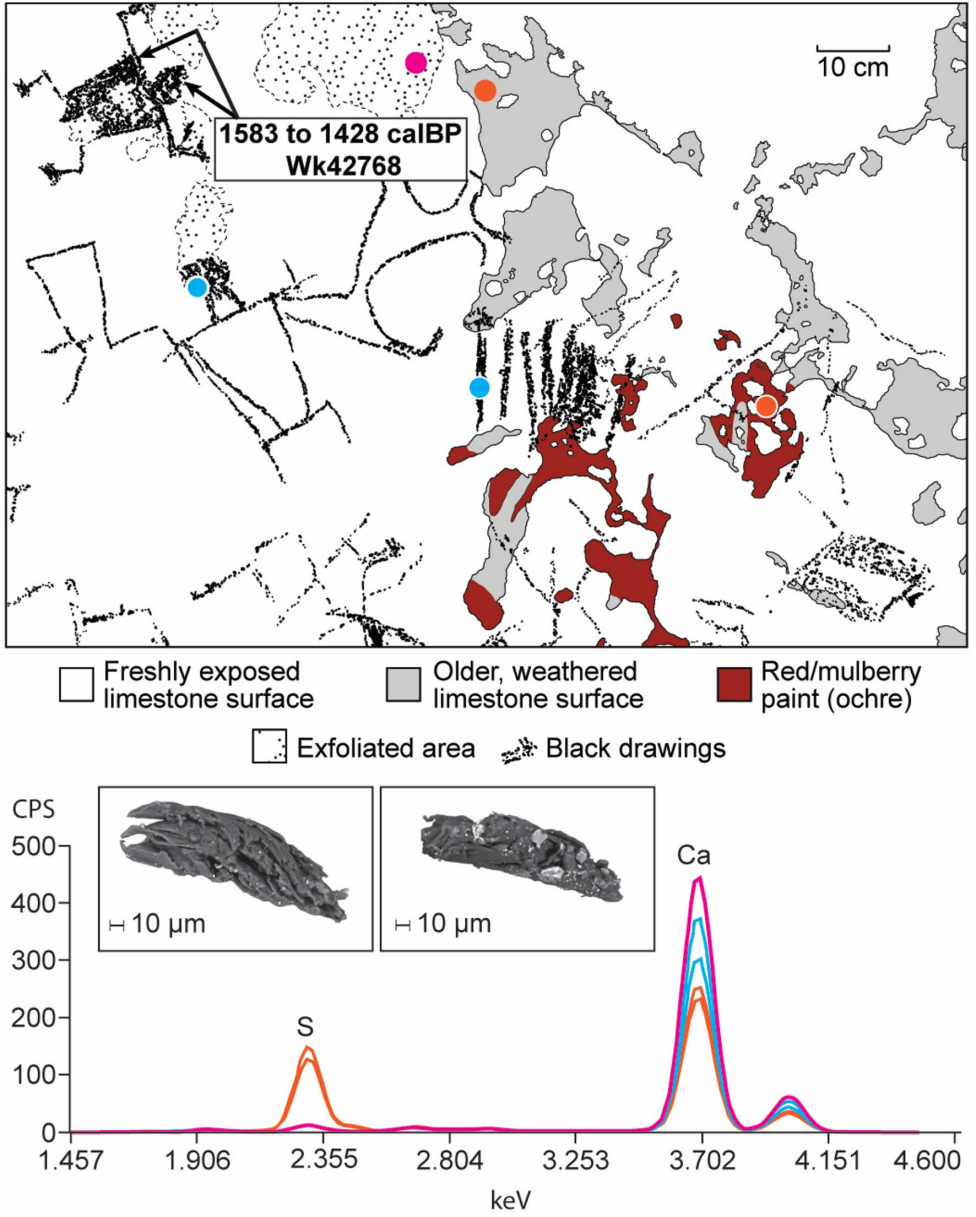
Ceiling art panel at Leang Bulu Bettue showing pXRF spectra indicative of geological salts (assay location shown with dots; relative element abundance in counts per second), the location of sampling for radiocarbon dating and (inset) scanning electron micrographs (secondary electron images taken at 10 kV) of charcoal grains collected from the dated anthropomorphic motif.

The island of Sulawesi lies at the centre of the Indonesian Maritime Continent (IMC), a region characterized by major ocean–atmosphere interactions^16,19^. The lowland ‘tower’ karst of Maros-Pangkep sits at the southern end of the Tonasa Formation, an isolated platform sequence of Eocene to late Miocene marine-deposited limestones dominated by photic zone benthic foraminifera^20^. The karst landscape covers an area of ~ 450 km^2^ between 4°7′S and 5°1′S and is situated on an alluvial plain close to the western shoreline of Sulawesi’s southwestern peninsula, bounded to the east by the volcanic mountains of the Western Dividing Range. The isolated karst towers and plateau-like hill masses of Maros-Pangkep range from 150 to 300 m in height and 1 to 10 km in diameter, with networks of footcaves, overhangs, and high-level cave passages^21^ housing an extensive rock art assemblage first reported by archaeologists in the 1950s^22^. More than 300 cave sites or rock shelters harbouring parietal imagery have been documented in these karsts, with new rock art sites found each year during ongoing field surveys (Fig. 1). At least two temporal phases of prehistoric imagery are evident based on direct-dating and studies of superimposition. The first phase, executed from at least 45.5 ka until ~ 20 ka based on Uranium-series dating of associated overlying coralloid speleothems^8–11^, is found exclusively on older case-hardened cave surfaces. Pleistocene rock art is characterised by red/mulberry hued pigments and typically comprises hand stencils and figurative paintings of large-bodied endemic land mammals (especially wild suids) shown in outline profile with irregular infill. The second, more recent rock art phase is found on fresher cave surfaces (or, more rarely, superimposed over the older cave surfaces and art styles) and is assumed to be only a few thousand years in age. This phase is attributed to the Neolithic farming communities colloquially known as ‘Austronesians’ who colonised Sulawesi ~ 4000–2000 years ago. This younger art phase is usually executed in black pigment (charcoal^21^) and is characterised by small images of anthropomorphic figures and domesticated fauna such as dogs, as well as an array of geometric motifs and abstract signs/symbols. We conducted direct Accelerator Mass Spectrometry (AMS) radiocarbon (^14^C) dating of a charcoal drawing of a human figure, typical of the Austronesian-style, located on the fresher surfaces of the ceiling panel at Leang Bulu Bettue. This yielded an age of 1583‒1428 calBP (SHCal 2020, Wk42768); to our knowledge this is only the second absolute age determination reported for such a motif^23^ (Fig. 2).

**Figure 3:**
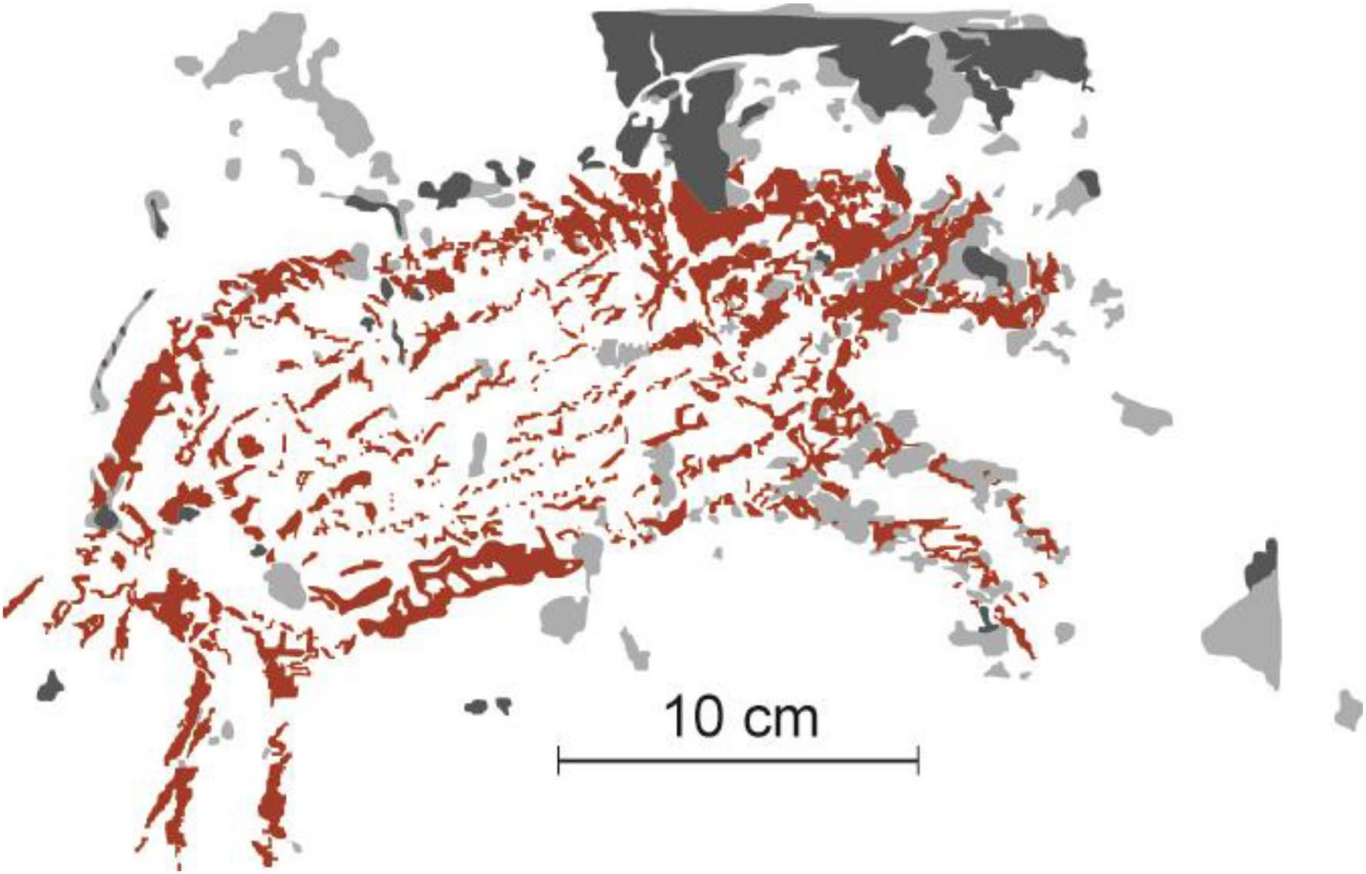
Rate of salt-induced exfoliation affecting a figurative painting of a suid. This rock art motif is located at Leang Pattae, a limestone cave open to the public at Taman Prasejarah Leang-Leang, Maros-Pangkep. The artwork is undated but it was executed in the same artistic style used to depict animals during the Late Pleistocene rock art phase. Dark grey shading highlights the exfoliated areas as documented in 1950^21^. The light grey highlights exfoliated areas as documented in 2013.

**Figure 4:**
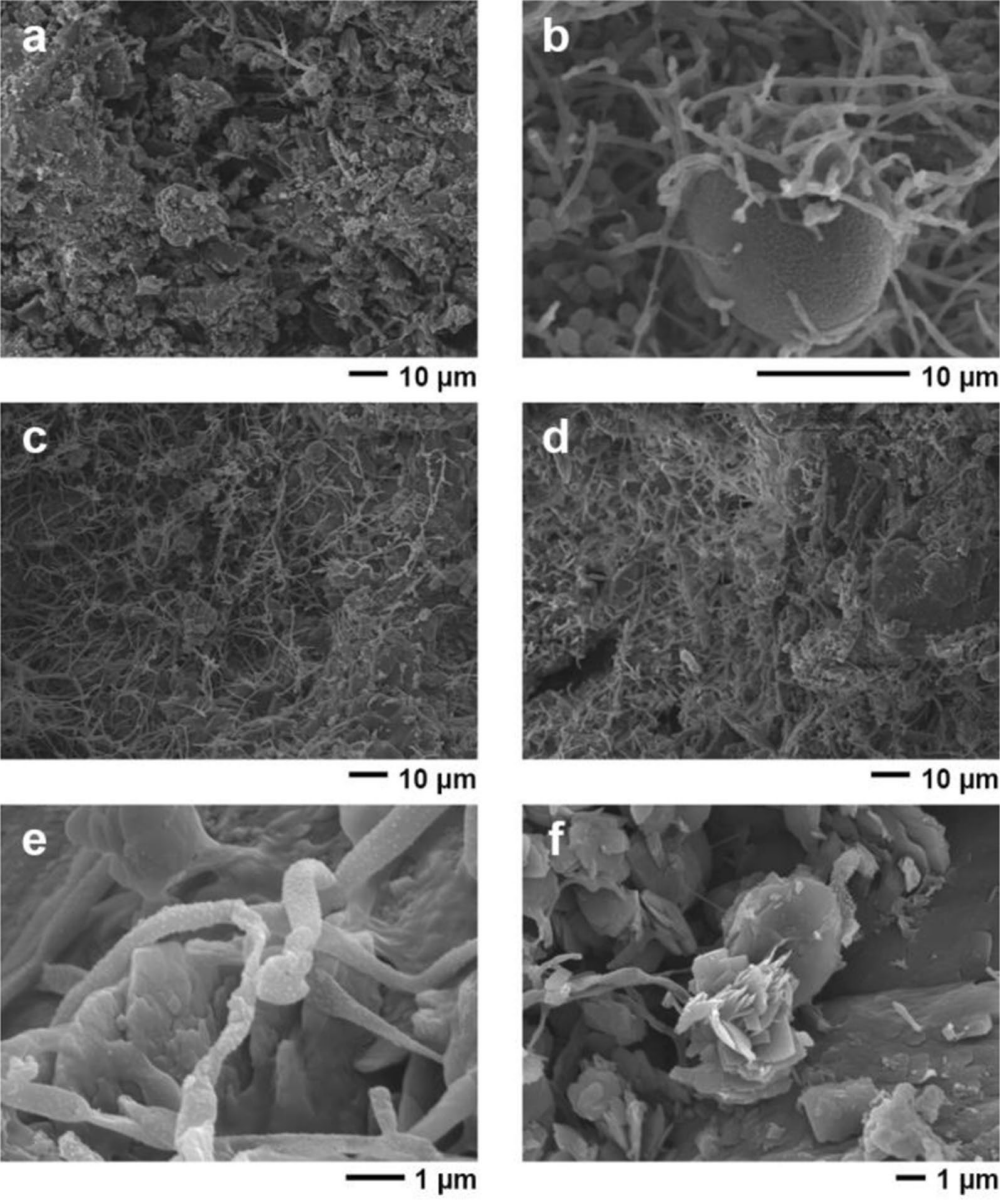
Scanning electron micrographs (secondary electron images taken at 10 kV) of microbial matts on the panel surfaces at (**a**) Leang Balang Pakalu 5; (**b**) Leang Timpuseng; (**c**) Leang Balang Pakalu 5; (**d**) Leang Timpuseng; (**e**) Leang Lompoa; and (**f**) a typical gypsum rosette crystal structure, also from Leang Lompoa.

In almost all sites containing early art, the hand stencils and figurative motifs are heavily affected by exfoliation of the limestone cave wall/ceiling surfaces that comprise the artists’ “canvas” (Fig. 3 and Supplementary Information—SI). The deposition of solutes (chiefly irons, silicones, and calcium carbonates) from the bedrock and surrounding environment concentrate and oxidise at the limestone’s surface, blocking pores to form a mineralised rind—a process known as case-hardening^24^. The case-hardened surfaces of the Maros-Pangkep caves, co-created by biofilms including plentiful microbial mats^25–28^ (Fig. 4), regulate water penetration on limestone surfaces, preventing rapid moisture uptake^29^ (p. 217). While case-hardening makes the surface layer more resistant to weathering, the sub-surface zone immediately underneath is weakened by loss of cement matrices, the void spaces becoming susceptible to the accumulation of evaporites such as geological salts, especially where the outer crust has been breached^24,29^. From the earliest published descriptions of the Maros-Pangkep rock art over half a century ago, exfoliation of the older case-hardened surface of panels has been noted—termed “blistering” in early reports^22^ (p. 30). Also evident is the execution of the second phase of art production on more recent surfaces and/or overlying small areas of remnant case-hardening. In many instances, such as at Leang Bulu Bettue, Austronesian style rock art was created on long-exfoliated cave surfaces that preserve residual hand stencils of presumed Late Pleistocene age, suggesting that in some cases the early rock art was already in a state of advanced deterioration several thousand years ago. Similarly, at the cave art site of Leang Lambatorang Dutch graffiti signed with dates as early as AD 1769 is visible on “fresh” limestone surfaces exposed by exfoliation of early phase rock art (SI). A mounting body of quantitative and anecdotal evidence suggests that the rate of exfoliation is increasing (SI). Indonesian cultural heritage professionals, local academic archaeologists, and site custodians from nearby communities (some of whom have worked at the rock art sites long-term, even intergenerationally) report that the destruction of rock art through spalling has accelerated, with more panel loss from exfoliation over recent decades than at any other time in living memory^8,11^.

Studies of human–environment interactions suggest changing climates were key factors in the evolutionary emergence of our species in Africa^30–32^. In addition to being influenced by our environments, humans have had a measurable impact on their surroundings since the Pleistocene epoch, with general acceptance that human activity is *the* dominant global cause of environmental change^33^ (p. 171). Human-induced environmental changes have accelerated in the Holocene with the compound, cumulative effects of agriculture (including rice cultivation in Asia from 6.5 ka and on Sulawesi from 3 ka), colonial migration and expanded human settlement (the late 1400 s onwards), industrialisation (1760s onwards), nuclear and thermonuclear testing (1940s on), and today’s persistent greenhouse gas emissions^19,33–35^. Although the Pleistocene rock art of Maros-Pangkep has existed throughout tens of thousands of years of climate variation in response to the earth’s orbital geometry, there is no doubt that global carbon cycling and greenhouse gasses are now significantly amplifying climatic responses^36^ (p. 24), and, we infer, hastening the deterioration of the unique, irreplaceable record of early human artistic culture in a little-understood region, one that continues to provide important insights into the culture of the first peoples of IMC^8–11,21^.

The tropics have always been integral to the global climate system, essentially acting as a “heat engine” driving millennial-to centennial-scale oscillations^36^. The Australasian monsoon domain within which Sulawesi is located (from the equator to 10° S latitude and from 110° E–130° E longitude) is the most atmospherically dynamic region on Earth^19,37^. Monsoon intensity has been the dominant feature of the Australasian paleoclimate^37^ (p. 110) with recent regional reconstructions beginning to disentangle the varied, localised responses to climate fluctuations across the IMC^16,19,36^ (p. 22). Generally speaking, a dry background climate state dominated the Australasian monsoon region during the Late Pleistocene (40–12 ka). At the time the earliest rock art in Maros-Pangkep was being created (at least 45–35 ka), climatic conditions were cooler and moister/more humid, resulting in lower evaporation^36^ (pp. 26–27). This cool trend increased from ~ 32 ka throughout the glacial period when the tropics experienced generally stable dry conditions, peaking at the Last Glacial Maximum (LGM) 22–18 ka as a result of sea levels dropping to at least ~ 125 m lower than today, reducing Sea Surface Temperatures (SST) by 1–3 °C and constricting the Indo-Pacific Warm Pool (IPWP). Current evidence suggests the monsoon disappeared entirely over northern Australia at this time, but there were at least periodically wet conditions over the IMC^36^ (pp. 27–28). The monsoon appears to have undergone a process of reinvigoration (with some reversals) from 15 ka and was well-developed by 13–12 ka; oxygen isotope data throughout the tropics records relatively stable temperatures, even during the Arctic Cool Reversal (14.5 to 12.5 ka) and Younger Dryas (12.85–11.65 ka)^37^ (p. 102), before a return to generally wetter, warmer conditions across the IMC with the inundation of the Sahul Shelf to the south 12–11 ka, and the Sunda Shelf to the west ~ 9.5–8.5 ka, warming SSTs and increasing evaporation^19,36,37^.

Though less frequent or extreme than it is today, the El Niño Southern Oscillation’s (ENSO’s) El Niño phase became a dominant climate feature 7–5 ka^36^ (p. 30)^37^ (p. 103). The IPWP reached thermal maximum 6.8–5.5 ka when coccolith records from the Banda Sea east of Sulawesi suggest a significant shift in the position of the monsoon (~ 6 ka) owing to low-latitude insolation forcing^37^. Localised aridity from El Niño characterises much of Australasia from 5 ka^36^ (p. 29)^37^ (pp. 103, 109), though more extreme and longer dry events are recorded 2.5–1.7 ka^37^ (p. 103). Modelling of ambient temperatures over the last millennium suggests peak pre-industrial warmth in Australasia 0.8–0.65 ka^38^ (p. 5581). Terrestrial and marine records show dry El Niño dominated conditions on Sulawesi over the past 400–300 years^39^ coincident with the advent of rice cultivation in the south of the island, dating to at least the fourteenth century^40,41^, though rice has been grown in West Sulawesi for more than 3 ka^35^. Minimum temperature anomalies are recorded at the time of the Northern Hemisphere’s so-named ‘Little Ice Age’ in the 16^th^ and early nineteenth centuries^38^ (p. 5381). These data complement stable isotope records from the Great Barrier Reef of Australia that document ENSO intensifying from the early 1600 s, with extended droughts in the mid-1760s to 1780s^37^ (concurrent with dated Dutch graffiti at Leang Lambatorang—SI). During these drier conditions, extensive deforestation of the fertile plains in Maros-Pangkep is likely to have occurred in step with the emergence of the earliest agrarian kingdoms (complex chiefdoms) in South Sulawesi in the late thirteenth century, and certainly from the fifteenth century onwards with the rise of the Makasar Goa-Talloq kingdom^41^. More extreme wet and dry years occur in the current era, which many scientists refer to as the ‘Anthropocene’ (< 1965)^37^ (p. 103)^33,34^, precisely because of the prevalence of human induced environmental change. There is a clear trend toward more frequent ENSO activity in recent decades compared with past centuries or millennia^16^ (SI). These more intense and prolonged dry climate cycles, interspersed with moister recharge from monsoonal rainfall—combined with abundant evaporative water sources in the inundated rice fields at the base of the tower karsts and brackish aquaculture ponds toward the coastline—are providing highly favourable conditions for the deposit of the evaporite salts causing exfoliation of ancient cave/shelter surfaces.

The extensive shorelines of the IMC make the region sensitive to climate change^16,42^, with Indonesia identified as a high-risk area in terms of drought-induced food security and extreme weather events such as tsunamis, flooding, and bush fires impacting densely populated coastal areas^3,4,18^. The specific physiogeography of the Maros-Pangkep region makes it highly susceptible to ENSO. Located in the IPWP, Sulawesi’s hydroclimate is influenced by the changing position of the Intertropical Convergence Zone (ITCZ) and varying Indo-Pacific Walker circulation, with rainfall highest on the western coast compared with the rest of the island^43^. The highly seasonal precipitation in Maros-Pangkep is intensified by the central mountain range dividing the southwestern peninsula or ‘arm’ of Sulawesi, which preferences rainfall from southward monsoon migration—delivering ~ 80% annual precipitation December-March, while preventing astral winter rainfall from reaching the region as the ITCZ migrates north^19,44^ (p. 11). Weather station data from Sulawesi shows 13 El Niño events between 1972 and 2012 with dry days (rainfall of > 1 mm) increasing by more than two months in El Niño years compared with typical La Nina conditions^43^. Rainfall observations between 1967 and 2015 indicate that southwest Sulawesi is most highly affected by El Niño, experiencing longer dry spells, and, in the case of the Maros-Pangkep region, the most consecutive dry days^45^. It is this combination of seasonal rainfall with alternating prolonged dry conditions under El Niño that provides ideal conditions for salt efflorescence and other forms of evaporite deposition.

**Figure 5:**
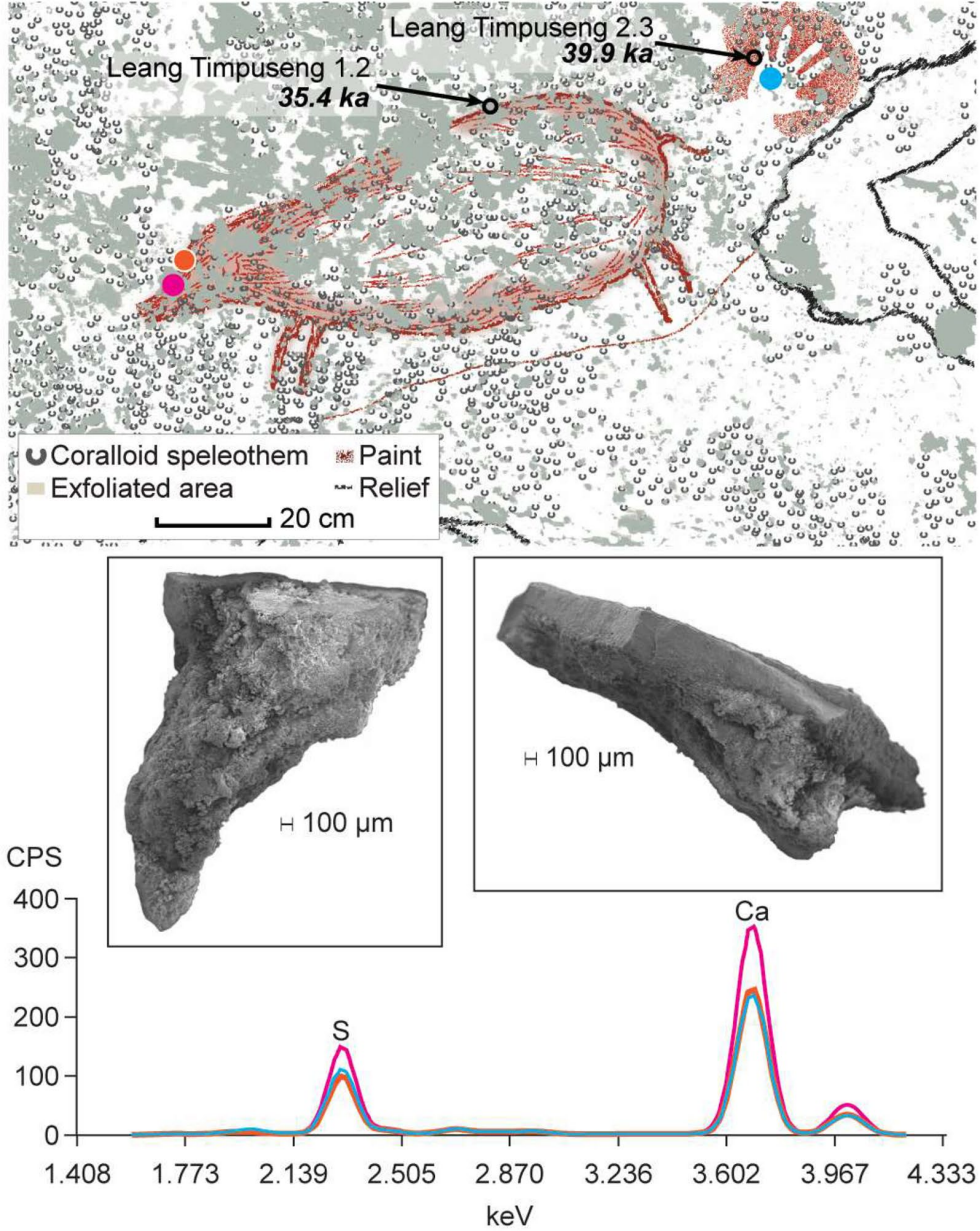
Ceiling art panel at Leang Timpuseng showing pXRF spectra indicative of geological salts (assay location shown with dots; relative element abundance in counts per second), the location and age of Uranium series dates^7^ and (inset) scanning electron micrographs (secondary electron images taken at 10 kV) showing the extent of haloclasty/salt crystallisation on the underside (left) and profile (right) of the same spall flake.

In an effort to determine why the early rock art of Maros-Pangkep is disappearing so rapidly, we investigated 11 cave art sites in these limestone karsts (Fig. 1), including Leang Timpuseng, which houses the oldest known hand stencil in the world (minimum age of 39.9 ka)^9^ (Fig. 5). We found ubiquitous evidence for salt efflorescence, including a clear physiochemical distinction between surviving residual case-hardened panel surfaces that house the Late Pleistocene mulberry/red hand stencils and irregularly infilled animal motifs, and fresher surfaces that contain the younger, Austronesian style black drawings (Fig. 2).

Salt efflorescence is the growth of salt crystals deposited by saline solution, also known as haloclasty or salt crystallisation. When the solution evaporates, crystals form, expand, and contract as the environment heats and cools, causing repetitive strain. When heated, geological salts such as gypsum, sodium sulphate, sodium carbonate, magnesium sulphate and calcium chloride can expand by three or more times their initial size, while deliquescent salts like sodium chloride dissolve and recrystalise as relative humidity waxes and wanes^46,47^. Cycles of temperature (especially periodic heat) and humidity induce salt migration, precipitation, crystal growth, hydration, and expansion, triggering mechanical stress on stone surfaces at both the “macro” level (e.g., flaking/exfoliation) and on a “micro”’ scale in the form of granular disintegration. Beginning at the solute/air interface, high relative humidity and/or high ambient temperatures trigger precipitation and evaporation, causing salt crystals to grow, filling pore and void spaces in stone until successive crystallisation pressure generates cracks; crystallisation continues in the form of columns which lift spall flakes, detaching them from the surface^29^ (p. 227) as we observed, for instance, at Leang Timpuseng (Fig. 5).

Elevated sulphur abundances were recorded throughout the 11 Maros-Pangkep rock art panels examined (Figs. 2, 5 and SI), except in areas of active water wash over the panels, something also observed in similarly ancient rock paintings in nearby East Kalimantan (Indonesian Borneo)^10^. We identified salts typical of haloclasty on the underside of spall flakes; specifically, gypsum (calcium sulfate) (× 2) and halite (sodium chloride) at Leang Lompoa, Leang Balang Pakalu 5 and Leang Timpuseng (SI). These salts epitomize different aspects of the weathering processes: flaking and granular disintegration. With hydration, gypsum can increase in volume by 63%, creating accompanying swelling pressure, while the dehydration (phase transition to anhydrite) induces a 39% volume decrease^29^ (p. 228); however, halite, a deliquescent salt found on the surfaces at Leang Timpuseng, has a smaller hygrometric morphology that tends to form a granular crust on damp substrates^29^ (p. 227). Notably, Leang Timpuseng is one of two studied sites in closest proximity to the extensive wet rice fields (*sawah*) characteristic of Maros-Pangkep. The identification of halite at Leang Timpuseng is also important because the higher the concentration of sodium chloride in solutes, the lower the temperature required for hydration/dehydration transitions (shrink and swell) of calcium sulphide crystals that catalyse the flaking/spalling of rock surfaces^29^ (p. 230). This indicates a far greater risk for accelerated degradation of Pleistocene rock art in proximity to *sawah* and also *empang*—the extensive networks of saline aquaculture ponds in the lower reaches of the Rammang-Rammang River, the watershed boundary at the heart of the Maros-Pangkep region.

Global warming has already impacted natural and human systems to varying degrees of severity as greenhouse gas emissions raise global temperatures an average of ~ 0.2 °C per decade. Unabated, conservative predictions are for global warming to 1.5 °C above pre-industrial (> 1750) levels within decades (i.e., between 2030 and 2052), with areas of climate sensitivity such as the tropics set to experience up to 3 × higher temperature increases. This trend in higher global temperature accompanies increased frequency and intensity of extreme weather events in the mid-latitudes (i.e. the tropics) where hot days have already increased by ~ 3  ͦC^17^. Here we have outlined the increasing deterioration of the Maros-Pangkep rock art assemblage as a consequence of climate change over at least the past four centuries, with apparent rapid acceleration over the past 40 years associated with anthropogenic climate forcing. The challenges facing the preservation of Pleistocene rock art in the tropics are being accelerated by climate change, with urgent action now required. If sustained net zero human CO^2^ emissions were reached (and non-CO^2^ reduced elevating radiative forcing) it may still be possible to halt anthropogenic global warming on a multi-decadal timescale^17^. The dramatic CO^2^ emission reductions achieved as a result of restricted mobility during global Covid-19 pandemic shutdowns show what is possible^1^.

Understanding the environmental context of early rock art is crucial for designing effective management strategies to help preserve these irreplaceable images from the human past^48,49^. Our study of the materiality of the Maros-Pangkep rock art panels shows that geological salts are ubiquitous, something borne out across the IMC by emerging physicochemical work on rock art in adjacent Indonesian islands^12–15,50^. Loss of the painted limestone “canvas” from salt efflorescence enhanced by El Niño conditions is the most pressing threat to rock art preservation in this region – aside from the industrial-scale quarrying of limestone (with associated pollution yet to be quantified)^8,51–53^. Long term physical and chemical monitoring has been ongoing in the Franco-Cantabrian rock art sites of Lascaux and Altamira since 1963 and 1997, respectively, with detailed climatic studies currently underway at Chauvet, Orgnac (Ardèche), Esparros (Hautes-Pyrénées), and Villars caves^54^. While a small-scale monitoring program has recently been initiated in Maros-Pangkep by the Makassar-based cultural heritage agency Balai Pelestarian Cagar Budaya, more support for this initiative is required, especially infrastructure investment. As some of the earliest known examples of artistic creativity on the planet, the Pleistocene rock art of Maros-Pangkep warrants monitoring and conservation efforts on par with those carried out over decades in Europe. The exceptionally old cave art of Indonesia is located within a dynamic tropical environment that renders it particularly vulnerable to the destructive impacts of climate change, adding unique urgency to this call for further research.

## Methods

### Field sampling

#### pXRF

Portable X-Ray Fluorescence (pXRF) assay locations were selected for features of interest presenting the pXRF aperture to minimise any air gaps and associated X-Ray attenuation. Assay were collected at the height of an El Niño induced drought in late 2015, with strong winds noted in the latter part of the field program. The exceptionally dry conditions were perfect for evaporite deposition and it is likely that the preceding hot, dry climatic period contributed to the ubiquity of geological salts on the cave/shelter surfaces.

#### Spall Flake removal

Small (< 2.5 cm diameter), thin flakes that were near spalling were collected from the case-hardened cave surfaces for use in laboratory-based analyses 2017. At Leang Timpuseng the art panel is on the ceiling. Spall flakes from the edge of the ceiling art panel near its junction with the back wall of the rockshelter were collected. At Leang Lompoa and Leang Balang Pakalu 5 spall flakes from the rock art panels, including black and red pigments from motifs (SI), were collected.

### Physicochemical characterisation

#### pXRF

Pigment characterizations were undertaken using a Brucker TitanS1 800 pXRF instrument, equipped with a silicon drift detector, Rh target X-ray tube (maximum voltage 50 kV, default to 150 °C with ultralene window) and five position motorized filter changer. Two beam phases collecting for 90 analytic seconds were taken for each assay (180 s per spectrum). Phase 1 parameters: 45 kV, 10.45 µA with a Ti 25 µm, Al 300 µm filter in the beam path. Phase 2 parameters: 15 kV, 31.55 µA without a filter. Relative element abundances are derived from the manufacturer’s empirical parameters calculations corrected for limits of detection (2 σ errors doubled and subtracted from the calculated parts per million readings to remove any spurious concentrations). Spectra were additionally manually interrogated using Bruker Artax software (V8). Certified reference materials were used as internal standards for the dataset to monitor instrument stability^21^.

#### Scanning electron microscopy

Field emission SEM was undertaken on a Joel 7100 JSM. Energy Dispersive Spectrometry (EDS) of relative element abundances measured in spot, area and element mapping modes using an Oxford X-Max 80 probe and Aztec software. Sections of the collected spall flakes were mounted on stubs (face-up, face-down and in cross section where possible) and gold coated to facilitate the analysis of both the case-hardened outer surfaces and the underlying salt covered surfaces being mechanically pushed free of the limestone massive.

#### Synchrotron powder diffraction

Fragments of the spall flakes were crushed into homogenized powders manually using an agate mortar and pestle. Once powdered, they were placed into 0.3-mm-diameter borosilicate capillaries and mounted on the Australian Synchrotron beamline. Diffraction data were collected at the at a wavelength of 0.77412(3) Å, calibrated using a NIST SRM 660b, from 5° to 85° 2Theta, with a Mythen microstrip detector with an inherent step size of 0.002°, using two detector positions and a collection time of 5 min per position. Samples were rotated at around 1 Hz during data collection to ensure good powder averaging. Phase identifications were undertaken using Panalytical Highscore with the ICDD PDF4 database^10^.

#### Radiocarbon dating

Black pigment samples were removed from the rock face using a sterile blade, with material collected directly into clean Teflon vials. Pigment from two locations within the same motif, demarcated subsamples LBB 3 (shoulder) and LBB 4 (head) (Fig. 2), were collected in the hope that enough pigment could be harvested to produce two age determinations acting as internal verification for the motif age. Unfortunately, the pigments were thin and only a single target could be generated. The AMS radiocarbon determination was made at the University of Waikato Radiocarbon Dating laboratory (Wk-42768) with the sample washed in hot 10% HCl, rinsed and air dried prior to analysis. Calibration was undertaken using OxCal software (v4.4.2)^55^ applying atmospheric data from Hogg et al. 2020^56^.

## Supplementary Information

Supplementary Information 1.

Supplementary Information 2.

## Data Availability

Data are available in the Supplementary Data.
