# Supplementary material for: The effects of climate change on the Pleistocene rock art of Sulawesi
Source: Sci Rep. 2021 May 13;11:9833. doi: 10.1038/s41598-021-87923-3 (PMC8119963; doi:10.1038/s41598-021-87923-3)
Supplement: Supplementary file 1 — Supplementary Information 1. [file 41598_2021_87923_MOESM1_ESM.pdf]

Supplementary Information

The effects of climate change on the Pleistocene rock art of Sulawesi.

J. Huntley[1]*, M. Aubert[1, 2], A. A. Oktaviana[1, 3], R. Lebe[4, 5], B. Hakim[5], B. Burhan[2], L. Muhammad Aksa[4], I. Made Geria[3], M. Ramli[4], L. Siagian[7, 8], H. E. A. Brand[9] and A. Brumm[2].

1 PERAHU, Griffith Centre for Social and Cultural Research, Griffith University, Gold Coast, Queensland, Australia, 2 Australian Research Centre for Human Evolution, Environmental Futures Research Institute, Griffith University, Brisbane, Queensland, Australia, 3 Pusat Penelitian Arkeologi Nasional (ARKENAS), 4 Balai Pelestarian Cagar Budaya, Makassar, Indonesia. 5 Faculty of Humanities, Hasanuddin University, 6 Balai Arkeologi Sulawesi Selatan, Makassar, 7 Museum Kepresidenan Republik Indonesia, Balai Kirti, 8 Universitas Gadjah Mada (Fakultas Ilmu Budaya-Magister Arkeologi),
9 Australian Synchrotron, Clayton, Victoria, Australia.
*e-mail: j.huntley@griffith.edu.au

SI Data: Tables are provided in the attached Excel file:

Table SI 1: pXRF chemical element relative abundances measured on the Pleistocene rockfaces of art panels in Maros-Pangkep;

Table SI 2: Summary of U-series age determination for rock art panels included in this study; and

Table SI 3: Chemical abundances measured via SEM-EDS.

SI Sections (this document):

Figure SI 1.1 Rapid panel loss (spalling) at Leang Jarie *Page SI 2*

Figure SI 1.2 Dutch graffiti at Leang Lambatorang *Page SI 3*

Figure SI 1.3 Ceiling art panel at Leang Bulu Bettue and Leang Timpuseng *Page SI 2*

Two Review of paleoclimatic conditions in the Australasian monsoon domain *Page SI 4*

Three Observed increases frequency and intensity of ENSO in Australasia *Page SI 13*

Four Review of physicochemical studies of rock art surfaces in the IMC *Page SI 16*

Five Powder Diffraction Patterns and interpretation *Page SI 18*

Six SEM-EDS mapping of slats spall flake from Leang Timpuseng *Page SI 25*

Seven Statistical modelling of pXRF assays *Page SI 26*

Eight pXRF Assay Locations *Page SI 28*

SI SECTION ONE.

Supplementary Figures

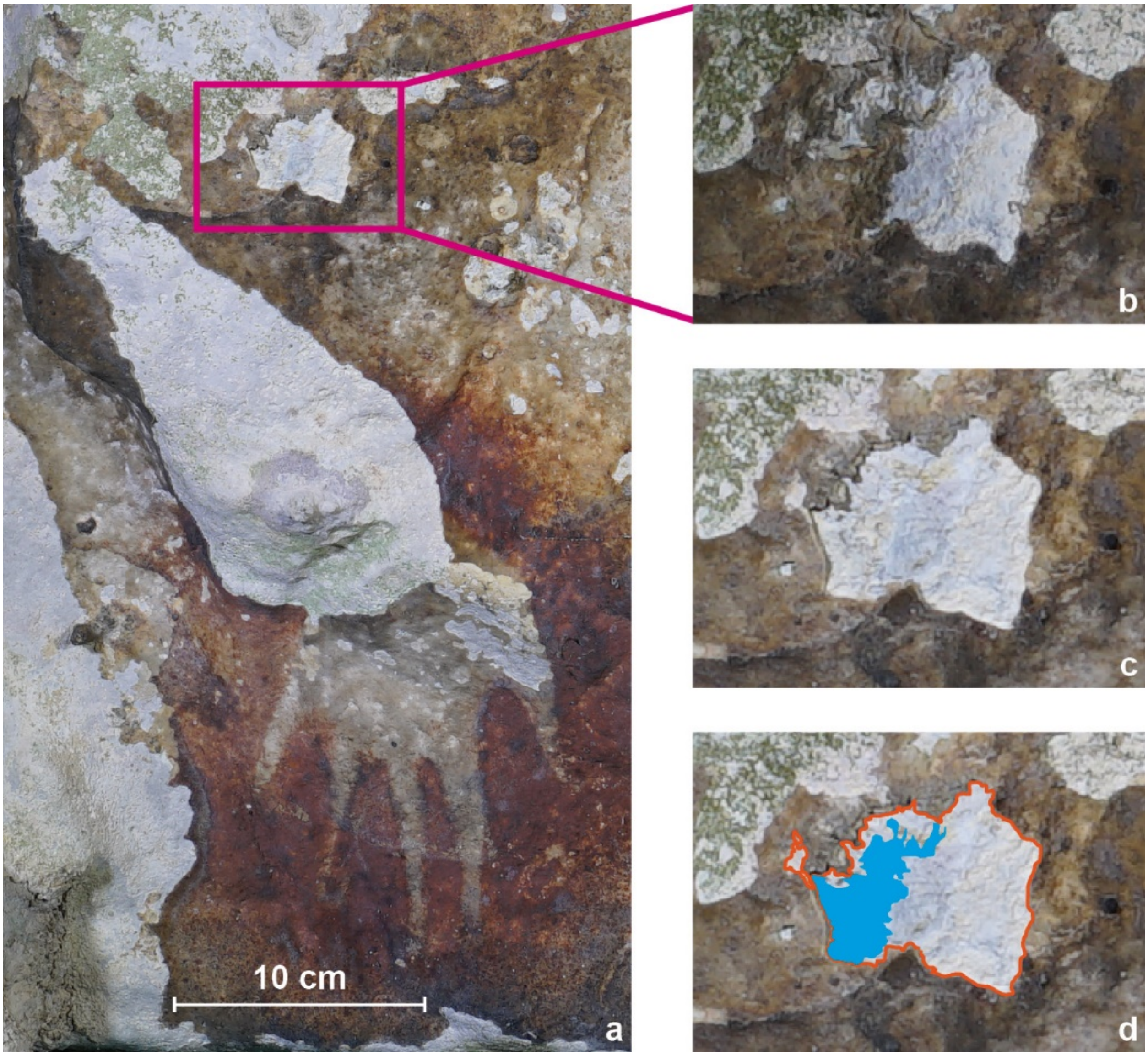

Figure SI 1.1 Rapid panel loss (spalling) at Leang Jarie **a)** overview of the art panel; **b)** spall scar in October 2018; **c)** spall scar in March 2019; **d)** overly of panel surface loss over just five months. Photographs Balai Pelestarian Cagar Budaya, Sulawesi Selatan, supplied by Rustan Lebe.

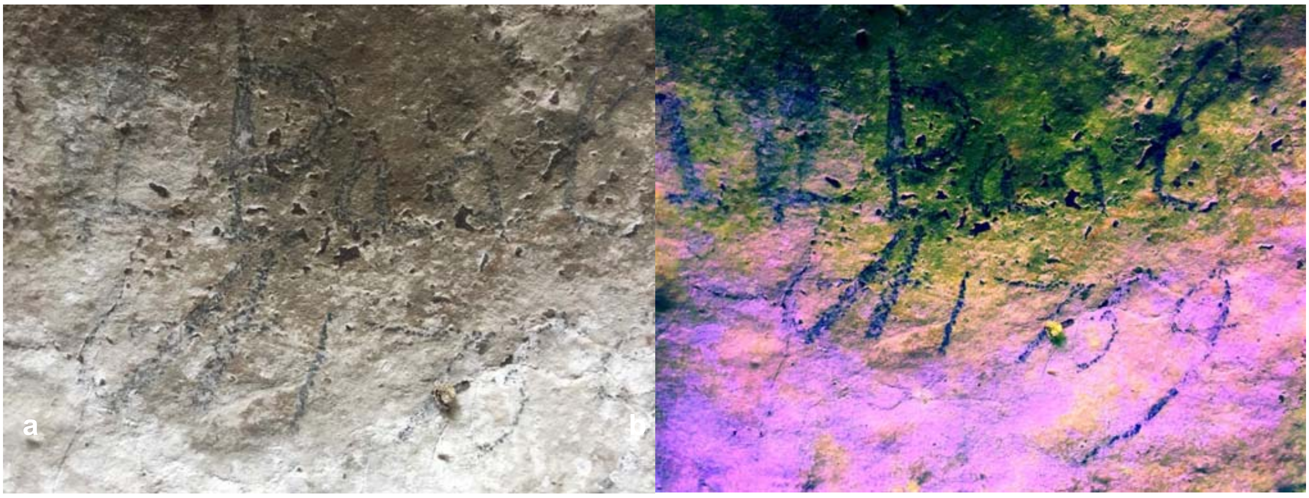

Figure SI 1.2 a) Dutch graffiti dated 1759 at Leang Lambatorang; **b)** with D-stretch enhancement. Photographs Adam Brumm.

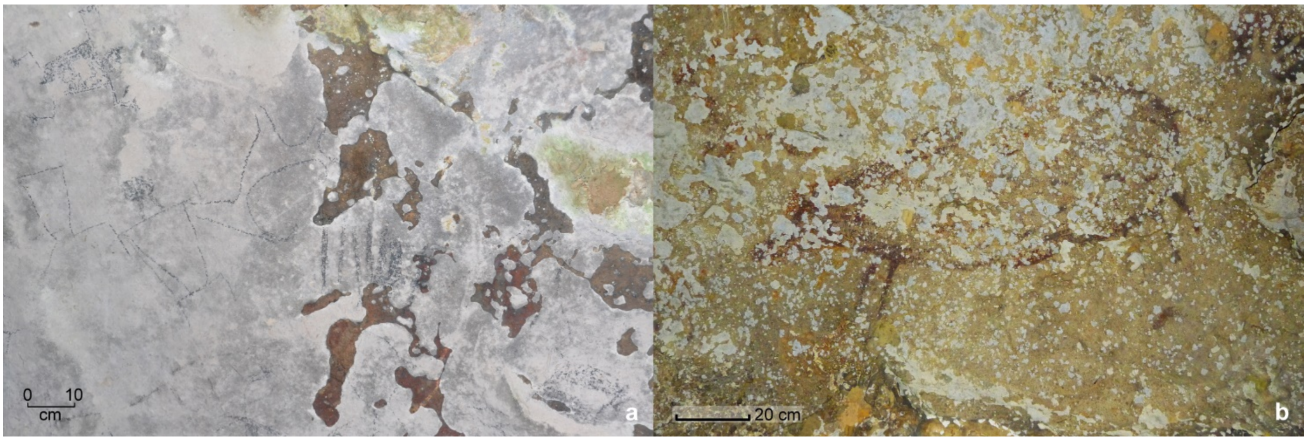

Figure SI 1.3 Photographs of art panels illustrated in Figures 2 and 5. a) Ceiling art panel at Leang Bulu Bettue; b) Ceiling art panel at Leang Timpuseng. Photographs Maxime Aubert.

SI SECTION TWO.

Review of Paleoclimatic conditions in the Australasian monsoon domain.

The tropics are, and have always been, a key component of the global climate system. They act as its 'heat engine', driving millennial to centennial scale climate change. The size and intensity of the Indo-Pacific Warm Pool and associated Indonesian Throughflow, both located on the southern edge of Sulawesi, are closely linked to El Niño Sothern Oscillation (ENSO) and the Indian Ocean Dipole[1:98-99] meaning numerous interconnected factors and effects are expressed in seasonal precipitation patterns. Indeed, the Australasian monsoon domain is the most atmospherically dynamic region on earth[2]. Over the 45, 000 years (hereafter 45 ka) timescale that rock art has been produced in Indonesia, the earth's orbital geometry has been the biggest external influence of climate, with global carbon cycling and greenhouse gasses now amplifying climatic responses across a variety of systems[3: 24]. Only very recently have regional climate proxy datasets begun to illuminate the varied, localised responses of Indonesia (and the rest of the maritime continent) to the complex of interconnected inputs driving climate variation. This section reviews the available evidence, summarising key climate variation events and their timing in the Australasian region, and where available southern Sulawesi. The time period incorporates major shifts including the end of Marine Isotope Stage 3, full glacial conditions, deglacial warming, through to establishment of more-or-less modern conditions in the late Holocene, including most recent centennial to decadal-scale variation and present ENSO cycles[3: 22].

The dominant feature of the paleoclimate of tropical Australasia over the past 40 ka is the varying intensity of the monsoon with insolation effecting its timing and penetration (especially at 35 ka, 21 ka, 11 ka and now) with changes in landmass:ocean ratios influencing regional moisture availability[1:110]. The heat of the equatorial tropics combined with complex climate drivers including fluctuating sea:land ratios, the Indo-Pacific Warm Poll, Indian Ocean Dipole, South

East Trade Winds, the Summer Monsoon and Indonesian throughflow make the Australasian region the most climatically dynamic on the planet (here from the equator to 10ºS latitude and from 110ºE-130ºE longitude)[2:22, 3]. Inter-decadal pacific oscillation modulates the frequency and intensity of ENSO impacts resulting in multi-decadal epochs that are either significantly direr or wetter than others[2: 23]. Paleoclimatic variation in precipitation can be more-or-less explained by the presence or absence of the monsoon, however temperature changes do not necessarily correlate with moister regimes leading to effective precipitation (that account for evaporation) are often out of step with major global temperature shifts[2: 29].

40-35 ka

Cooler temperatures than today with more moisture due to lower evaporation, leading into cool humid conditions[3:26-27, 4].

32-22 ka

Cooling began around 32 ka with glacial conditions in New Guinea by ~28 ka[3:27]. Moister availability reflected in the speleothem record of Borneo was relatively constant during the early glacial period tending to increased variability by 28-24 ka[1:108]. Terrestrial paleoclimate records from Flores and Java show a drying trend (particularly between 31-26 ka), while coeval records from Sumatra and Wet Papua remained relatively wet indicating localised regional responses[3:27. 1:103]. Glacial temperatures were reach ~28 ka with the tropics experiencing dry, stable conditions characterised by a close correlation between effective precipitation and regional insolation. Abrupt drying and increased climate variability are recorded in the speleothem record on Borneo 26.5-25 ka followed by an accelerated decent to cool conditions after 24 ka leading into the Last Glacial Maximum (LGM)[3:27].

22-18 ka (LGM)

Sea surface temperatures were 1-3ºC cooler in tropical waters at the LGM constricting the Indo-Pacific Warm Pool. Peak glacial extent in Australasia occurred ~19 ka, with moderate cooling in the tropical oceans owing to their thermal buffering capacity. Though vegetation records from Indonesia show cooler drier conditions with the retraction of woody rain forest species and expansion of grasses and herbs. The summer monsoon was not present in northern Australia during the LGM, however tropical regions to the north show at least periodically wet conditions[3:27-28]. Sea levels were around 125m lower than today reducing the proportion of ocean in the Australasian region to 65% (compared to modern conditions of 78%). Wind and rainfall patterns were quite different than modern conditions due to the weakened monsoon[1:100]. Pollen records throughout the lowlands of tropical Australasia reflect universally cooler direr conditions[1:108].

18-15 ka early deglacial

Sea surface temperatures increased rapidly between 18-15 ka, though there was a lag in the Indian Ocean beginning at 15 ka. Wetter warmer conditions are evidence in Indonesia generally ~17 ka, though on Borneo drier conditions persist until ~15 ka with a rapid shift to wetter conditions thereafter, except for a millennia long plateau centred around the Arctic Cold Reversal ~13.2 ka[1:104; 3;28].

15-12 ka late deglacial/terminal Pleistocene, Younger Dryas 12.85–11.65 ka and the Arctic Cool Reversal 14.5 to 12.5 ka.

Climatic conditions 15-12 ka were highly localised and variable with often conflicting responses across the Australasian region[3:28]. With the inundation of the Sunda Shelf, sea surface temperatures became warmer, probably increasing evaporation, thus re-invigorating of the

summer monsoon. Speleothem records from Borneo saw an increase in precipitation after 15 ka and then a reversal to somewhat drier conditions between 13.3 and 12.3 ka[1:104. 3:28]. Despite these variations it seems the summer monsoon was reinvigorated from 15 ka and well-developed by 13-12 ka. Oxygen isotope data from marine cores throughout the tropics indicate relatively stable temperatures during the Younger Dryas and the Arctic Cool Reversal – if anything the response of $\delta^{18}O$ in records ~14-12 ka reflects increased precipitation at this time[1: 102].

12-8 ka early Holocene

The early Holocene was the warmest period during the past 30 ka as a local insolation maximum coincided with the northern hemisphere thermal maximum[1:102]. Sea surface temperatures approached modern values 11-9 ka with land:sea ratio stabilising to modern conditions ~9.8 ka. While wetter conditions persisted in Indonesia 12-11 ka, modern vegetation patterns established by 11 ka[3:28].

8-5 ka mid Holocene

The mid-Holocene was characterised by peak warm and relatively wet conditions. Modern sea level and sea surface temperatures were reached by 8 ka with vegetation records in the north of Australasia showing a coeval change from grass-dominated to rainforest taxa. The thermal maximum of the Indo-Pacific Warm pool was reached 6.8-5.5 ka, though a warmer period was recorded 6.6-6.3 ka, peak wet condition recorded on Boreno ~5ka[1:103, 3;29]. Coccolith records from the Banda sea to the east of Maros suggest low-latitude insolation forcing due to a significant shift in the position of the monsoon ~6 ka[1:107].

5-0 ka late Holocene to present

Increased variability with enhanced direr conditions indicative of ENSO's El Niño phase/localised aridity are characteristic of much of Australasia from 5ka to present, with a stronger monsoon from ~4 ka[1:103, 109, 3:29]. El Nino's became a dominant feature from 7-5 ka as a result of a weakening Asian summer monsoon resulting weaker Walker Circulation and consequently weaker easterly trade winds over the tropical Pacific Ocean. This created increasingly favourable El Niño conditions resulting in more frequent El Niño events leading to increased fire activity and extended drought, interspersed by shorter, wet La Nina events – a climatic pattern that persists to today (though El Nino's were less frequent and less extreme than at present)[1:103, 3:30]. The Inter-tropical Convergence Zone governs moister regimes in Australasia bringing the summer monsoon rains and westerly winds (astral summer, November to March), in consort with local orographic influences this results in abundant seasonal rainfall in Indonesia for more than six months per year in non-El Niño years[3:98]. Extreme and longer el Niño events are recorded 2.5-1.7 ka[1:103]. Models of astral warm season (September-February) ambient temperatures in Australasia over the last millennium (1000-2001 CE) suggest peak pre-industrial warmth around 1150-1350 CE. From around 1350 a cooling trend that lasted several hundred years began. This cooling expressed as minimum temperature anomalies in the sixteenth and early nineteenth centuries, mostly coinciding with the peak of the Northern Hemisphere's Little Ice Age characterised by pronounced cooling that extended well into the tropical and subtropical regions of the Southern Hemispher[5:5381]. Most recently, there is a clear trend to post 1950 temperature increases with strong warming starting in 1095 CE and continuing to the present, attributed to increases in atmospheric greenhouse gasses. A recent detailed study of rainfall extremes from the past 40 years (1972-2012) in the Eastern Indonesian Maritime Continent (including three weather stations in Maros) shows strong correlation to ENSO, in particular a significant increase in days without rainfall in El Niño years

“…causing sever draughts in the region”[9:37]. Modelling of annual and seasonal maximum daily rainfall in Southeast Asia (1951-2007)[6].

Anthropocene/Modern climate

The Anthropocene (a synchronous global signature in geological forming materials that documents the profound and permeant effect humans have had on earth systems) is conservatively dated to late 1965 with the global fallout from nuclear bomb testing and use pronounced the event horizon marker[7]. The nuanced, regional responses of the eastern Indonesian maritime continent to human forced climate change within the Anthropocene are only starting to become apparent as more detailed regional paleoclimate and atmospheric studies become available.

Warmer seas surface temperatures and higher salinity are recorded in the stable isotope records of corals on the Great Barrier Reef of Australia, to the south, during the 18th century before cooling in the 19th and 20th centuries (specifically a cooling event was noted at 1870 CE at the end of the little ice age). The same records show variability in ENSO intensified from the early 1600s with extended droughts recorded in the mid-1760s to mid-1780s CE, followed by more extreme wet and dry years during the 20th century[1:103]. Modelling scenarios for the 21st century under global warming conditions predict increased monsoonal rainfall due to raising temperatures increasing atmospheric water vapor/weakening monsoon circulation[8:22].

The Maros region of southwest Sulawesi is highly susceptible to ENSO climate variation due to its physiogeography. Located within the IPWP, the hydroclimate of Sulawesi is influenced by changes in the position of the ITCZ and variation of the IPWC. The Maros region in particular has highly seasonal modern rainfall dominated by the AISM, intensified by the mountain range dividing the southwest handle of Sulawesi which preferences monsoon rainfall from the southward migration of the ITCZ - delivering ~80% of annual precipitation from December to March, while preventing the astral winter rainfall from reaching the region as the ITCZ migrates to the north. This strong

predisposition towards north-westerly AISM rainfall has been observed as making Maros ideal for tracking monsoon variation over long timescales[2, 9:11]. Shallow thermal convection in the pre-monsoon season in El Niño years lead to extremely dry conditions in ~October. Whereas prolonged wet conditions in La Nina years are associated with more transport of surface waters via the Indonesian throughflow in the Macassar Strait (on the southern shaw line of Sulawesi) resulting in prolonged moderately intense rainfall (rather than sever events which are more prevalent in transitional ENSO conditions)[10:39-40]. In addition, rainfall is higher on the western coast of Sulawesi compared to the rest of the island[11].

The northern Borneo paleoclimate records do not show modern monsoon seasonality, but instead record a region with year-round rainfall characterised by Western Pacific hydrology (apparently uncorelated to monsoon strength)[9:14]. Whereas records for south Sulawesi display strong astral summer rainfall seasonality that may mask millennia-scale boreal summer events recorded in the western Indonesian Maritime Continent[3:111].

The geographic location of the eastern Indonesian Maritime Continent in relation to the IPWP, IOD Indonesian throughflow and ITCZ, means convection is more active compared to other tropical regions. This area characteristically drier with more variation in precipitation, particularly around Sulawesi and the Maluku Islands, which are more susceptible to drought in astral winter via increasing consecutive dry days under El Niño conditions[10:37].

A dry background climate state dominated the Australasian monsoon region prior to the terminal Pleistocene (~12 ka). Sulawesi lies at the centre of sustained dry anomalies in rainfall records between 40-12 ka before a rapid shift to present seasonally high rainfall patterns in the Holocene. The two largest increases in rainfall coinciding with the flooding of the Sahul and Sunda shelves at ~12-11 ka and ~8.5 ka respectively[3:107].

Acronyms:

IPWP	Indo-Pacific Warm Pool
ITCZ	Intertropical Convergence Zone
IPWC	Indo-Pacific Walker circulation
ENSO	El Niño-Southern Oscillation
IOD	Indian ocean Dipole
AISM	Australian-Indonesian Summer Monsoon

SI Section Two, References Cited:

1. Reeves, J. M., H. C. Bostock, L. K. Ayliffe, T. T. Barrows, P. De Deckker, et al. (2013) Palaeoenvironmental change in tropical Australasia over the last 30,000 years–a synthesis by the OZ-INTIMATE group. *Quaternary Science Reviews* 74: 97-114.

2. Krause, C.E., M. K. Gagan, G. B. Dunbar, W. S. Hantoro, J. C. Hellstrom, et al. (2019) Spatio-temporal evolution of Australasian monsoon hydroclimate over the last 40,000 years. *Earth and Planetary Science Letters* 513: 103-112.

3. Reeves, J. M., T. T. Barrows, T.J. Cohen, A. S. Kiem, H. C. Bostock, et al. (2013) Climate variability over the last 35,000 years recorded in marine and terrestrial archives in the Australian region: an OZ-INTIMATE compilation. *Quaternary Science Reviews* 74: 21-34.

4. Kemp, J. and E. J. Rhodes (2010) Episodic fluvial activity of inland rivers in southeastern Australia: Palaeochannel systems and terraces of the Lachlan River. *Quaternary Science Reviews* 29(5-6):732-752.

5. Gergis, J., R. Neukom, A. J. Gallant and D. J Karoly (2016). Australasian temperature reconstructions spanning the last millennium. *Journal of Climate* 29(15): 5365-5392.

6. Villafuerte, M.Q. and J. Matsumoto 2015. Significant influences of global mean temperature and ENSO on extreme rainfall in Southeast Asia. *Journal of Climate* 28(5):1905-1919.

7. Turney, C. S.M., J. Palmer, M. A. Maslin, A. Hogg, C. J. Fogwill, et al. (2018) Global peak in atmospheric radiocarbon provides a potential definition for the onset of the anthropocene epoch in 1965. *Scientific Reports* 8(1): 3293, DOI:10.1038/s41598-018-20970-5.Konecky

8. Konecky, B., Russell, J. and Bijaksana, S. (2016) Glacial aridity in central Indonesia coeval with intensified monsoon circulation. Earth and Planetary Science Letters 437:15-24.

9. Kimbrough, A. K. (2016) The glacial-interglacial monsoon recorded by stalagmites from southwest Sulawesi, Indonesia. PhD Thesis, Australian Natioal Univerity: Canberra.

10. Lestari, S., J. I. Hamada, F. Syamsudin, J. Matsumoto and M D Yamanaka (2016) ENSO influences on rainfall extremes around Sulawesi and Maluku Islands in the eastern Indonesian maritime continent. *Sola* 12:37-41.

11. Giarno, D.D., A. A. Fisu and A. Mattingaragau (2020) Influence Rainy and Dry Season to Daily Rainfall Interpolation in Complex Terrain of Sulawesi. In *International Conference on Environment and Technology*. *IOP Conference Series* 469: 012003.

SI SECTION THREE.

Observed ENSO conditions and Climate Change Predictions in Australasia.

Data from weather stations recording precipitation on Sulawesi and the Mauku islands shows that the period 1972-2012 comprised 13 el Nino, 16 La Nina and 12 natural years[1:37-8]. Dry days tend to increase by more than three months in El Niño years. There is also an observed shift in wet season onset and withdrawal associated with ENSO transitional years[1:39].

El Niño years from the twentieth century onwards[2]: 20019-20, 2015-16, 2009-10, 2006-7, 2002-3, 1997-98, 1994-95, 1993-94, 1991-92, 1987-88, 1982-83, 1977-78, 1969-70, 1963-64, 1957-58, 1951-52, 1946-47, 1941-2, 1925-26, 1919-20, 1914-15, 1913-14, 1911-12, 1905-06, 1902-03 (Total 25).

La Nina years[3]: 2010-12, 2008-7, 1998-01, 1988-89, 1973-76, 1970-72, 1964-65, 1954-57, 1979-51, 1942-43, 1938-39, 1928-30, 1924-25, 1916-18, 1909-11, 1906-07, 1903-04 (Total 17).

Fire in Sumatra and Kalimantan from drought have been recorded in 1982-3, 1987, 1991, 1994, 1997, 2002, 2006, 2015 and were predicted for 2019/20[4].

Episodic, though regular, droughts implicating ENSO correlation were chronicled in 1528, 1596, 1707, 1756, 1839, 1891, 1894 CE at the northern edge of Southeast Asia, in Dayu Cave, in both dated historic records carved into the cave walls and correlated with stable isotope analyses of speleothems[5].

Natural disasters in the form of floods and drought are frequent in the Karos Maros-Pangkep region; in nine years between 2009 and 2018 five floods and four droughts were recroded[6]. The flood event in Maros in 2010 resulted from the La Nina which persisted well into 2011[7:225].

Climate predations modelled from present conditions (2004-2013) and Representative Concentration Pathway inputs (4.5 and 6.0)[8]. 2030s RCP 4.5 – slight rainfall decrease and would mean flooding at

current levels/frequencies; RCP 6.0 rainfall increase and an increase in flood severity and frequency, and over about double land surface area currently effected by floods. In reality, this is not likely one or the other – the region will probably experience combinations of the two (years of overall drier and then years of overall wetter conditions)[8:11]. Our research strongly suggesting increased seasonality under such conditions, with hotter daily temperatures & more consecutive dry days in dry seasons and then high rainfall/flooding with intense/extreme events in the wet seasons, would be highly detrimental to the preservation of Pleistocene cave/rockshelter surfaces in the Kast limestones.

The international disaster database records that of meteorological events related to climate change floods are most common, followed by drought[8:1]. Using the same methods with RCP 4.5 input, climate projections from 2018-2050 estimate a 17% increase in average rainfall when compared to today over the next 33 years[6: 3].

Policies for climate change adaptation in the Maros-Pengkep region include the intensification of rice growing to improve food security[6:8]. While more efficient, higher-yielding framing practices would be welcome, our research suggests that additional rice fields (increasing seasonal standing water storage) would be detrimental to the preservation of Pleistocene cave/rockshelter surfaces in the region. As the flood hazard venerability is defined primarily by topography[8:10], increased intensity and severity of rainfall events in our study are will almost certainly accelerate agents of deterioration as the low lying plains in from to the karts where rice production is ubiquitous, will be prone to flooding and water retention.

SI Section Three, References Cited:

1. Lestari, S., J. I. Hamada, F. Syamsudin, J. Matsumoto and M D Yamanaka (2016). ENSO influences on rainfall extremes around Sulawesi and Maluku Islands in the eastern Indonesian maritime continent. *Sola* 12:37-41.

2. Bureau of Meteorology 2019. El Niño - Detailed Australian Analysis, cited 16 August 2020: http://www.bom.gov.au/climate/enso/enlist/index.shtml

3. National Weather Service (2019). Climate Predication Centre, cited 23 October 2019: https://origin.cpc.ncep.noaa.gov/products/analysis_monitoring/ensostuff/ONI_v5.php

4. Field, R.D., G. R. Van Der Werf, T. Fanin, E. J. Fetzer, R. Fuller, et al. 2016. Indonesian fire activity and smoke pollution in 2015 show persistent nonlinear sensitivity to El Niño-induced drought. *Proceedings of the National Academy of Sciences* 113(33): 9204-9209.

5. Tan, L., Cai, Y., An, Z., Cheng, H., Shen, C.C., Breitenbach, S.F., Gao, Y., Edwards, R.L., Zhang, H. and Du, Y., 2015. A Chinese cave links climate change, social impacts, and human adaptation over the last 500 years. *Scientific reports*, *5*, p.12284.

6. Kemitraan (Partnership) (2019). Sustainable Livelihood and Ecoenterprise in Karst Ecosystem for Adapting to Climate Change, Project/program porosal ti The Adaptation fund, Washington D.C,cited 11 September 2020: https://www.adaptation-fund.org/project/sustainable-livelihood-and-ecoenterprise-in-karst-ecosystem-for-adapting-to-climate-change/

7. Arias-Ruiz, C., Elliot, M., Bézos, A., Pedoja, K., Husson, L., Cahyarini, S.Y., Cariou, E., Michel, E., La, C. and Manssouri, F., 2017. Geochemical fingerprints of climate variation and the extreme La Niña 2010–11 as recorded in a Tridacna squamosa shell from Sulawesi, Indonesia. *Palaeogeography, Palaeoclimatology, Palaeoecology*, *487*, pp.216-228.

8. Barkey, R., M. Nursaputra, M. F., Mappiase, M. Achmad, M. Solle and M. Dassir (2019). Climate change impacts related flood hazard to communities around Bantimurung Bulusaraung National Park, Indonesia IOP Conf. *Ser. Earth Environ. Sci*, *235*: 1-12.

SI SECTION FOUR.

Review of physicochemical studies of rock art surfaces in the IMC.

Previous pigment characterisations at Leang Sumpang Bita 2 in Pangkep regency identified gypsum on the surface of panels containing hand stencil motifs[1]. While ongoing monitoring work within the Maros-Pankep rock art assemblage has found consistently high CaO and SO_3 measurements indicative of gypsum at Gua Jing, Barugaya Cave, Uluwae Cave, Lambatorang Cave[2].

Detailed analysis of panel surfaces in neighbouring Kalimantan (Indonesian Borneo) clearly shows the presence of geological salts, particularly gypsum, overlying red and mulberry paintings[3, their SI]. This was born out by further analyses on rock art pigments in the region that also recorded gypsum on art panel surfaces[4, 5] and (as we have here) ubiquitous peaks of sulfur and calcium indicative of such surface geological salts[5].

Recent reports of a minimum of 16 hand stencil motifs in Lene Hara Cave, in Timor-Leste have been inferred as being of Pleistocene antiquity as excavated evidence for human occupation at the site dates to 43/41, 000 years ago[6:1]. Similarly to the Maros-Pangkenp rock art assembled, the researchers noted that on Timor-Leste hand stencils are the oldest part of the rock art sequence and that only occur only on the remaining older weathered “crust” surfaces. Whereas a latter superimposed sun/star motif overlies both the exfoliated and stencilled crust surfaces of the cave wall. Also, that the APT style motifs are exclusively on the exfoliated fresher surfaces[6:8]:

> “The stencils were applied on a mineral crust which itself directly overlays the limestone bedrock…However, natural weathering and heavy exfoliation has resulted in the loss of variable – but often significant – proportions of the crust, and in some places, it has disappeared altogether leaving many of the stencils heavily fragmented...” [6:5].

While these authors note that “the crust”/older cave surfaces upon which the extant hand stencils are found are “gypsum-based”[5:3, 7], it is far more likely that the physiochemical evidence they observed is salt efflorescence underneath the case-hardened cave surfaces, as we report in this article.

SI Section Four, References Cited:

1. Kurniawan, Robi, Grandprix Thomryes Marth Kadja, Pindi Setiawan, Basran Burhan, Adhi Agus Oktaviana, Budianto Hakim, Maxime Aubert, and Adam Brumm. (2019) Chemistry of prehistoric rock art pigments from the Indonesian island of Sulawesi. *Microchemical Journal* 146: 227-233.

2. Thosibo, A., N. H. Soekamto, A. Duli, and Y. Mulyadi. Broken painting in the prehistoric cave and chemical content of paint used in Maros Regency, South Sulawesi. In *Journal of Physics: Conference Series*, vol. 1341, no. 3, p. 032047. IOP Publishing, 2019.

3. Aubert, M., Setiawan, P., Oktaviana, A.A., Brumm, A., Sulistyarto, P.H., Saptomo, E.W., Istiawan, B., Ma'rifat, T.A., Wahyuono, V.N., Atmoko, F.T. and Zhao, J.X. (2018). Palaeolithic cave art in Borneo. *Nature*, *564* (7735): 254-257.

4. Ilmi, M.M., Nurdini, N., Maryanti, E., Saiyasombat, C., Setiawan, P. and Kadja, G.T.M., (2020) Multi-analytical Characterizations of prehistoric rock art pigments from Liang Karim Cave, Sangkulirang-Mangkalihat site, East Kalimantan, Indonesia. *Microchemical Journal*, p.104738.

5. Nurdini, N., Maryanti, E., Ilmi, M.M., Setiawan, P., Saiyasombat, C. and Kadja, G.T., (2020) Physicochemical investigation of prehistoric rock art pigments in Tewet Cave, Sangkulirang-Mangkalihat Site, East Kalimantan-Indonesia. *Journal of Archaeological Science: Reports*, *31*: p.102345 (1-11).

6. Standish, Christopher D., Marcos García-Diez, Sue O'Connor, and Nuno Vasco Oliveira (2020) Hand stencil discoveries at Lene Hara Cave hint at Pleistocene age for the earliest painted art in Timor-Leste. *Archaeological Research in Asia*: 100191, doi.org/10.1016/j.ara.2020.100191.

SI SECTION FIVE.

Powder Diffraction Patterns and interpretation of geological salts.

Worst-case scenario projections for green-house-gas emissions and associated climate change are becoming the likely trajectories, resulting in more drastic climate variation and stronger magnitude decay consequences for cultural heritage[1:20]. Depending on the pore/void spaces of the base geology (case-hardening) and the morphology & orientation of salts, crystals can exert pressure while not completely filling the pore space[2, 3:115, 3]. Where case-hardened surfaces are present, pressure from crystal growth can be catastrophic[4] (see their Figure 4: 8 for a schematic illustration of the development of case-hardening on limestone). Conservation professionals around the world have attributed increased humidity, changes in decohesion and accelerated cracking to a change in salt-crystallisation phenomena under variation in temperature and relative humidity as consequences of climate change on cultural heritage[1:11]. Sodium chlorides such as the Halite found on the panel at Lenag Timpuseng (below) are highly potentially detrimental to stone because they are very soluble and hygroscopic. They are the first salts to be dissolved during condensation of water from the surrounding air. Furthermore, halite in solution is mobile and penetrates and breaks up many crystalline structures when it recrystallizes. Halite is capable of retaining the adsorbed humidity in the masonry, it lowers the transformation temperature of hydrate salts and facilitates the transformation from one hydration state to another, i.e. anhydrite to gypsum[6].

Geological salts in and of themselves are not necessarily damaging to stone–they require the presence of water to become an aggressive agent of deterioration. That is to say, the source of the damage caused by geological salts is crystallization pressure, which originates from the growth of salt crystals in a supersaturated solution[2, 5]. Haloclasty or salt crystallisation causes rock disintegration by the seepage of saline solution into the cracks and joints of rock, water then evaporates leaving behind salt crystals. Once formed salt crystals expand when

heated, thereby exerting pressure on the confining rock. Slat weathering can also take the form of salt salutations decomposing limestone rocks to from sodium sulphate and/or sodium carbonate, again forming salt crystals once moistures evaporates[7:77]. Sodium sulphate, magnesium sulphate and calcium chloride are particularly harmful as their crystals can expand three times or more in size when heated. Salt efflorescence is generally associated with arid climates where strong heating causes strong evaporation[[8]].

The interconnected physics of the earth's systems such as enhancing evaporation, which can lead to an increase in the air's moister holding capacity–meaning more droughts, heatwaves and other secondary impacts (like more wildfires) from heat transfer when evaporation ceases, resulting in highly localised impacts from increasing climate hazards[9:1062]. Recent research demonstrates that humanity has already been impacted by climate hazards (draughts, floods, fires) that are predicted to intensify under a best-case scenario for global warming[9:1063].

We chose to undertake high resolution Synchrotron powder diffraction in order to confirm the inferred presences Gypsum ($CaSO_42H_2O$) via pXRF as Gypsum and Anhydrite ($CaSO_4$) have very different diffraction patterns. We also sought to describe the variety of minor phases of geological slats present to provide insights into the mineral transitions occurring due to temperature and humidity cycling.

Figure SI 5.1 Synchrotron Powder Diffraction Pattern - Leang Timpuseng

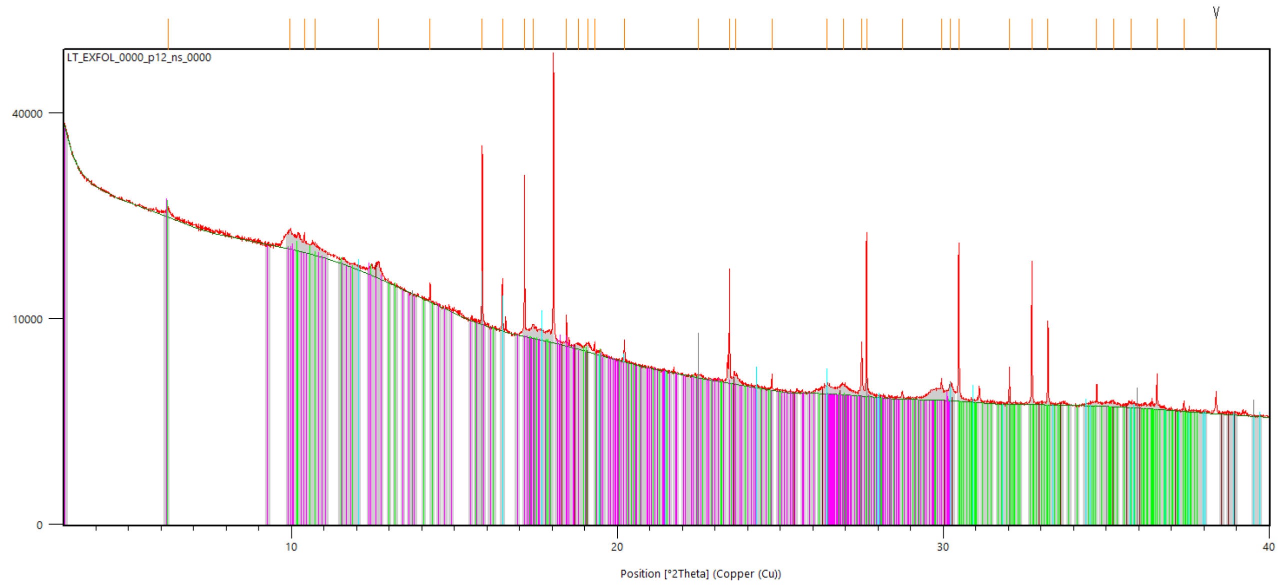

Green: Kaolinite $Al_2(Si_2O_5)(OH)_4$
Pink: Nacrite $Al_2(Si_2O_5)(OH)_4$
Blue: Hematite Fe_2O_3
Grey: Halite NaCl
Brown: Anatase TiO_2

Figure SI 5.2 Synchrotron Powder Diffraction Pattern - Leang Timpuseng Exfoliation Flake Replicate

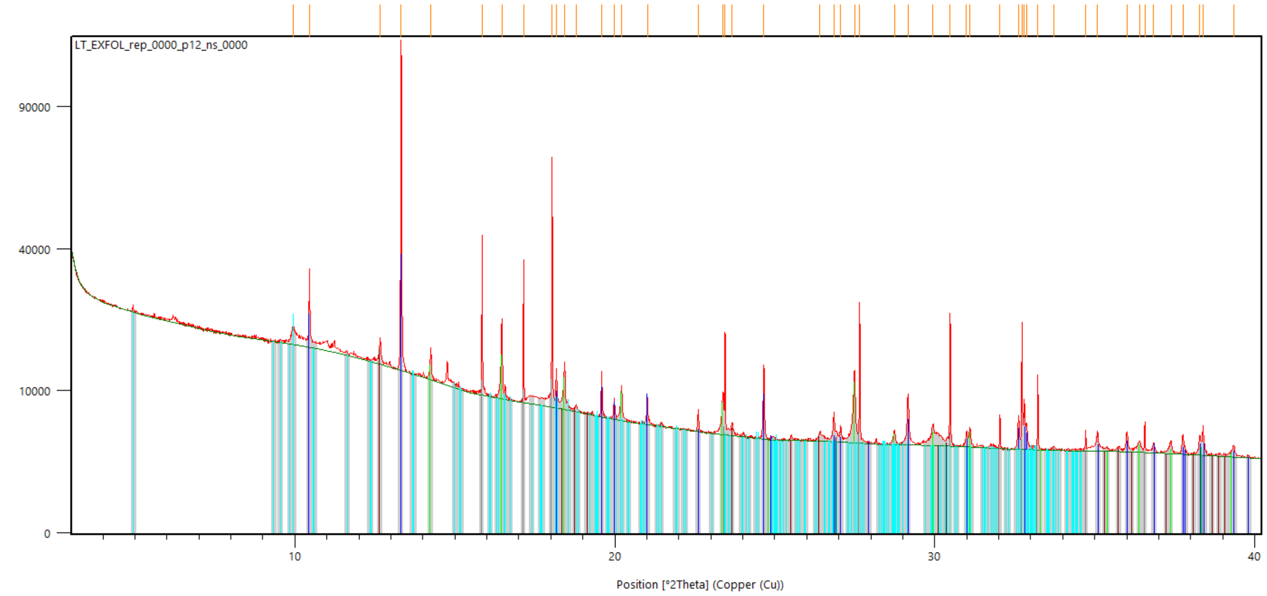

Blue: Quartz SiO_2
Green: Pyrite FeS_2
Turquoise: Chloritoid $(Fe^{2+},Mg,Mn^{2+})Al_2(SiO_4)O(OH)_2$
Grey: Zincite ZnO
Brown: Anatase TiO_2

Figure SI 5.3 Synchrotron Powder Diffraction Pattern - Leang Lompoa 1 (Black Paint)

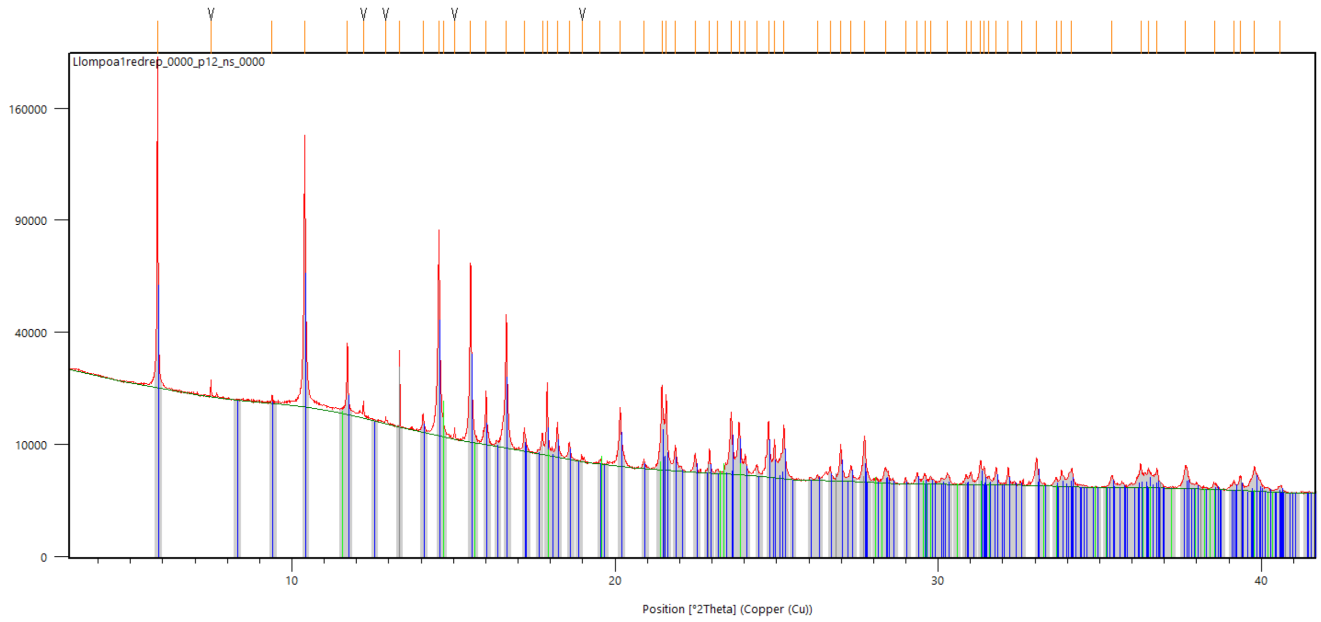

Blue: Gypsum $CaSO_4\ 2H_2O$
Green: Calcite $CaCO_3$
Grey: graphite C

Figure SI 5.4 Synchrotron Powder Diffraction Pattern - Leang Lompoa 2 (Back of the Exfoliation Flake)

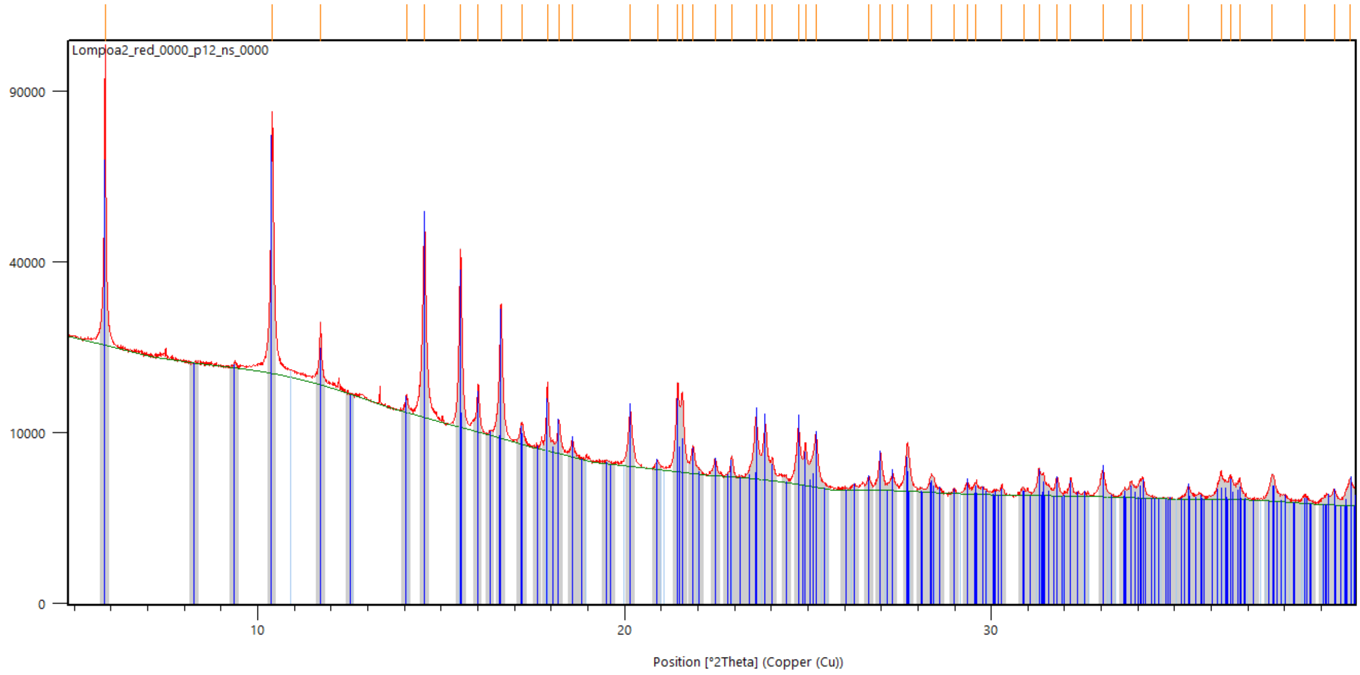

Blue: Gypsum $CaSO_4\ 2H_2O$

Figure SI 5.5 Synchrotron Powder Diffraction Pattern - Leang Balang Pakalu 5 Red Pigment

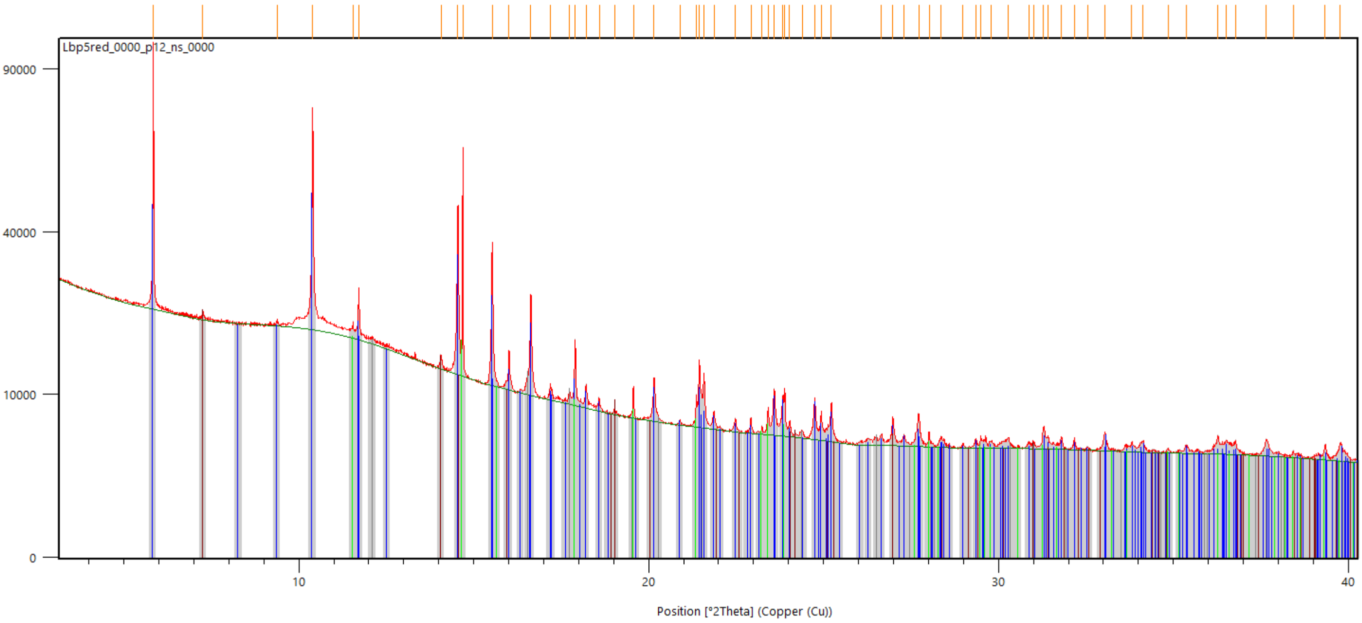

Blue: Gypsum	$CaSO_4$ $2H_2O$
Green: Calcite	$CaCO_3$
Grey: Hematite	Fe_2O_3
Brown: Bohmite	AlO(OH)

Figure SI 5.6 Synchrotron Powder Diffraction Pattern - Leang Balang Pakalu 5 Black Pigment

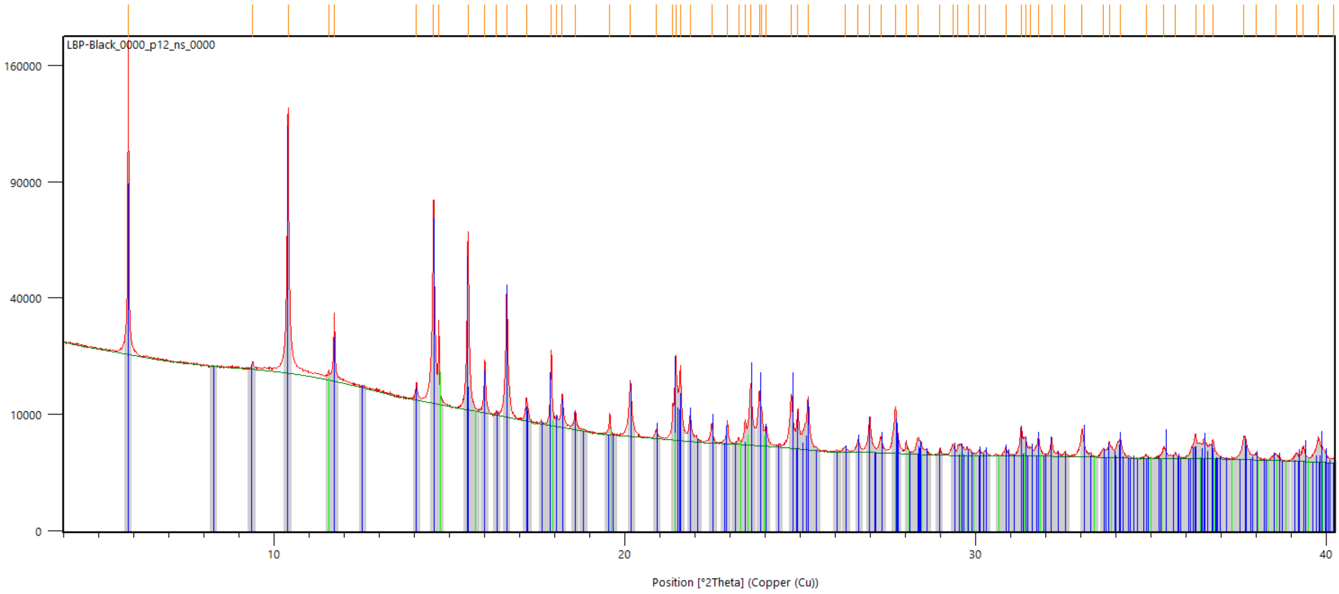

Blue: Gypsum	$CaSO_4$ $2H_2O$
Green: Calcite	$CaCO_3$

Figure SI 5.7 Synchrotron Powder Diffraction Pattern - Leang Balang Pakalu 5 Surface

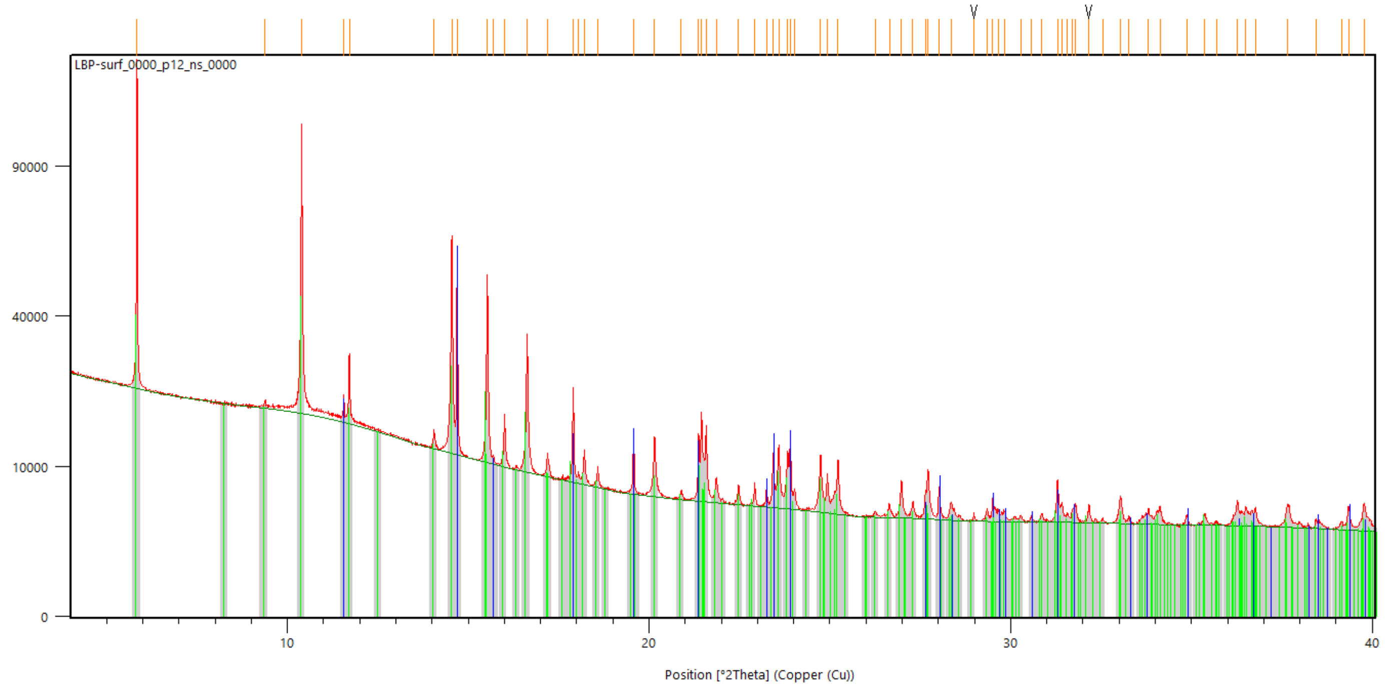

Blue is Calcite $CaCO_3$
Green is Gypsum $CaSO_4\ 2H_2O$

SI Section Five, References Cited:

1. Sesana, E., A.S.Gagnon, C. Bertolin and J. Hughes (2018) Adapting cultural heritage to climate change risks: Perspectives of cultural heritage experts in Europe. *Geosciences* 8(305)1-23.

2. Wittke, W., S. Sempreich and B. Plischke (1984) Felsmechanik; Grundlagen fu¨r wirtschaftliches Bauen im Fels. Berlin: Springer Verlag

3. Smith, B.J., P.A.Warke. and C.A. Moses (2000). Limestone weathering in contemporary arid environments: a case study from southern Tunisia. *Earth Surface Processes and Landforms* **25**: 1343 – 1354.

4. Wilhelm, K., H. Viles, E. Winter, Ó. Burke, S. Engelstaedter, and K. Z. Coyte (2017). Catastrophic Limestone Decay at the Central Sanctuary of Iupiter Dolichenus at Dülük Baba Tepesi in Southern Turkey: Causes and Implications for Future Conservation. *Conservation and Management of Archaeological Sites* **19, no. 1** : 3-29

5. Wüst, R.A. and Schlüchter, C., 2000. The origin of soluble salts in rocks of the Thebes mountains, Egypt: The damage potential to ancient Egyptian wall art. *Journal of Archaeological Science*, *27*(12), pp.1161-1172.

6 . Cooke, R. U. and I. J. Smalley (1968) Salt weathering in deserts. *Nature* 220: 1226–1227.

7 . Vasco, F (2010) Basic chemical mechanisms outdoors. Chapter 7, pp. 75-105 in, D. Camuffo, V. Fassina and J. Havermans (eds), *Basic Environmental Mechanism Affecting Cultural Heritage: Understanding deterioration mechanism for conservation purposes*, COST Action D: Chemical interactions between cultural artefacts and indoor environment (ENVIART): Nardini Editore, Firenze.

8 . Winkler, E. M. and E. J. Wilhelm, E. J. (1970) Salt burst by hydration pressures in architectural stone in urban atmosphere. *Geological Society of America Bulletin* 81: 567–572.

9 . Mora, C., D. Spirandelli, E. C. Franklin, J. Lynham, M. B. et al (2018) Broad threat to humanity from cumulative climate hazards intensified by greenhouse gas emissions. *Nature Climate Change* 8(12): 1062-1071.

SI SECTION SIX.

SEM-EDS mapping of geological slats at Leang Timpuseng.

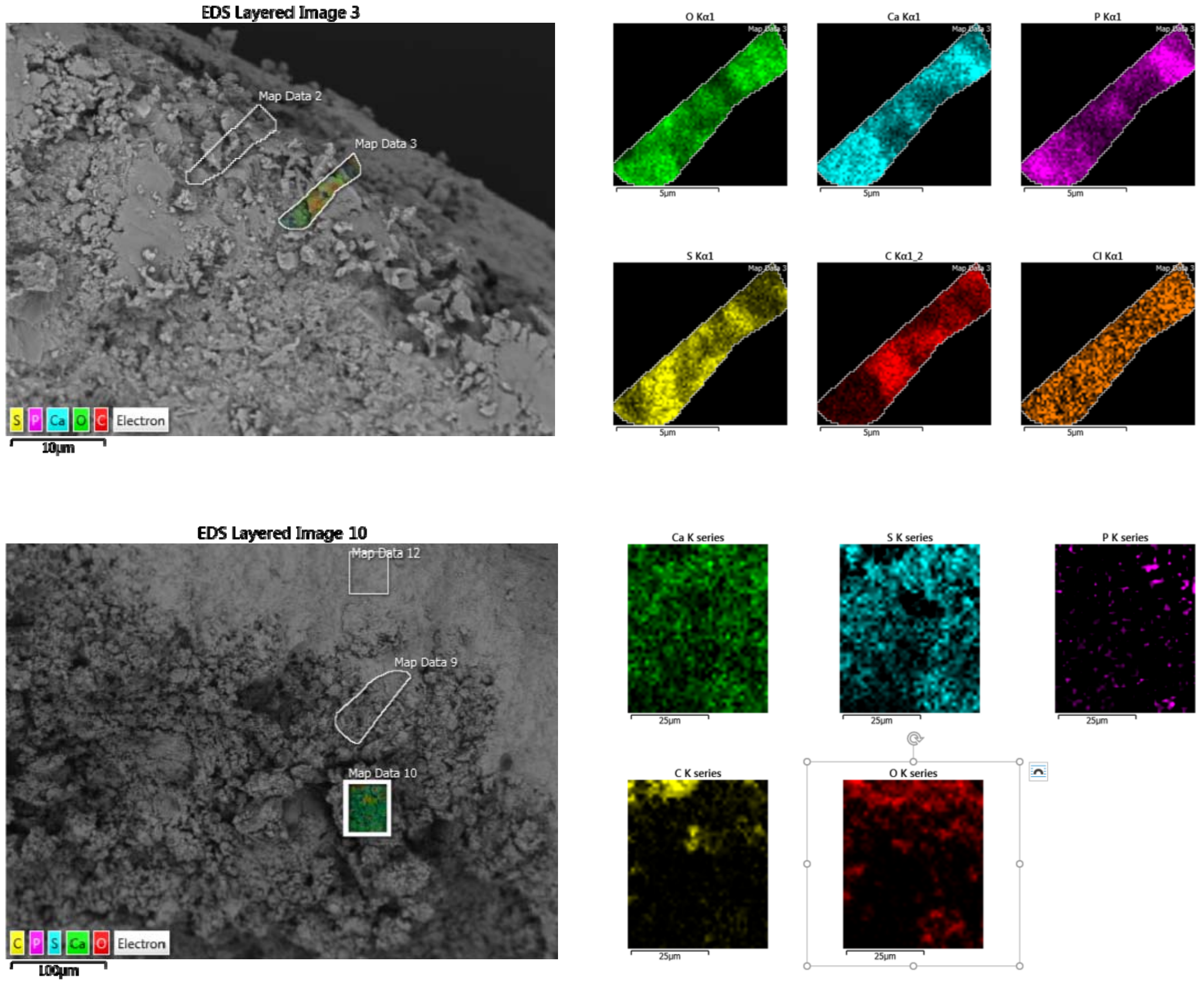

Figure SI 6.1 SEM-EDS mapping of geological slats on the underside of spall flake, Leang Timpuseng.

SI SECTION Seven.

Statistical modelling of pXRF assays.

Below: pXRF measured relative abundances for Al, Si, P, S, K, Ca, Ti, V, Cr, Mn, Fe, NI, Cu, Zn, As, Cd, Sn, and Pb are modelling in JMP13 software, using Principle Components Analysis (PCA) based on a correlation matrix.

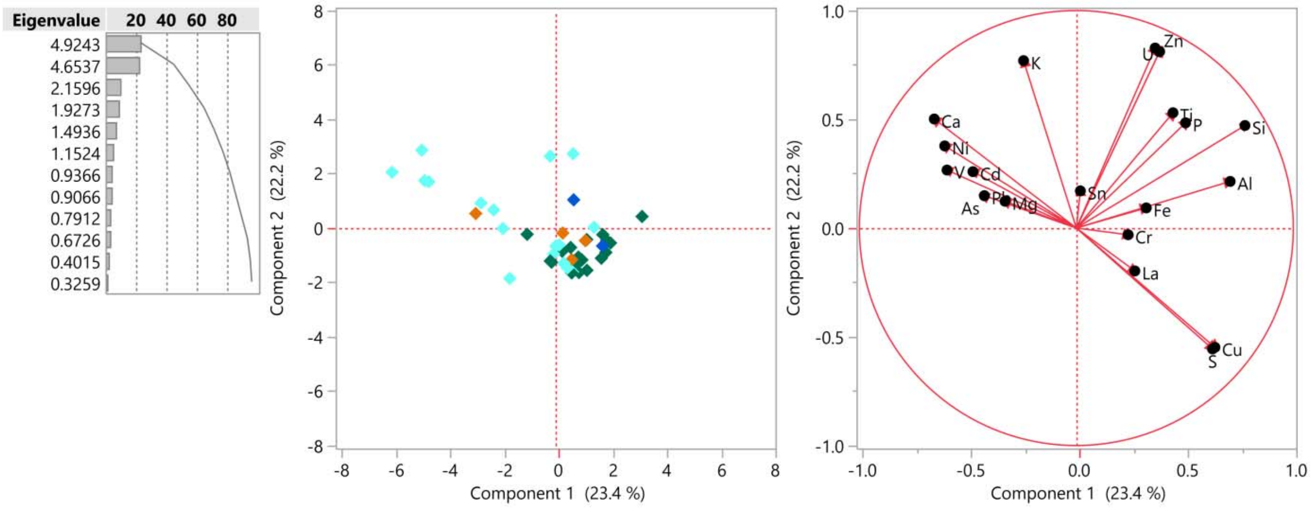

Figure SI 7.1 PCA loadings. Light blue - spall; Dark blue - fresh wash; Green - casehardening; Orange - casehardening with iron staining. Data from 10 of the 11 study sites: Leang Timpuseng, Leang Lompoa, Gua Jing, Leang Sampeang, Leang Pettakere, Leang Pattae, Leang Bulu Bettue, Leang Barugayya, Leang Sakapao and Leang Jarie.

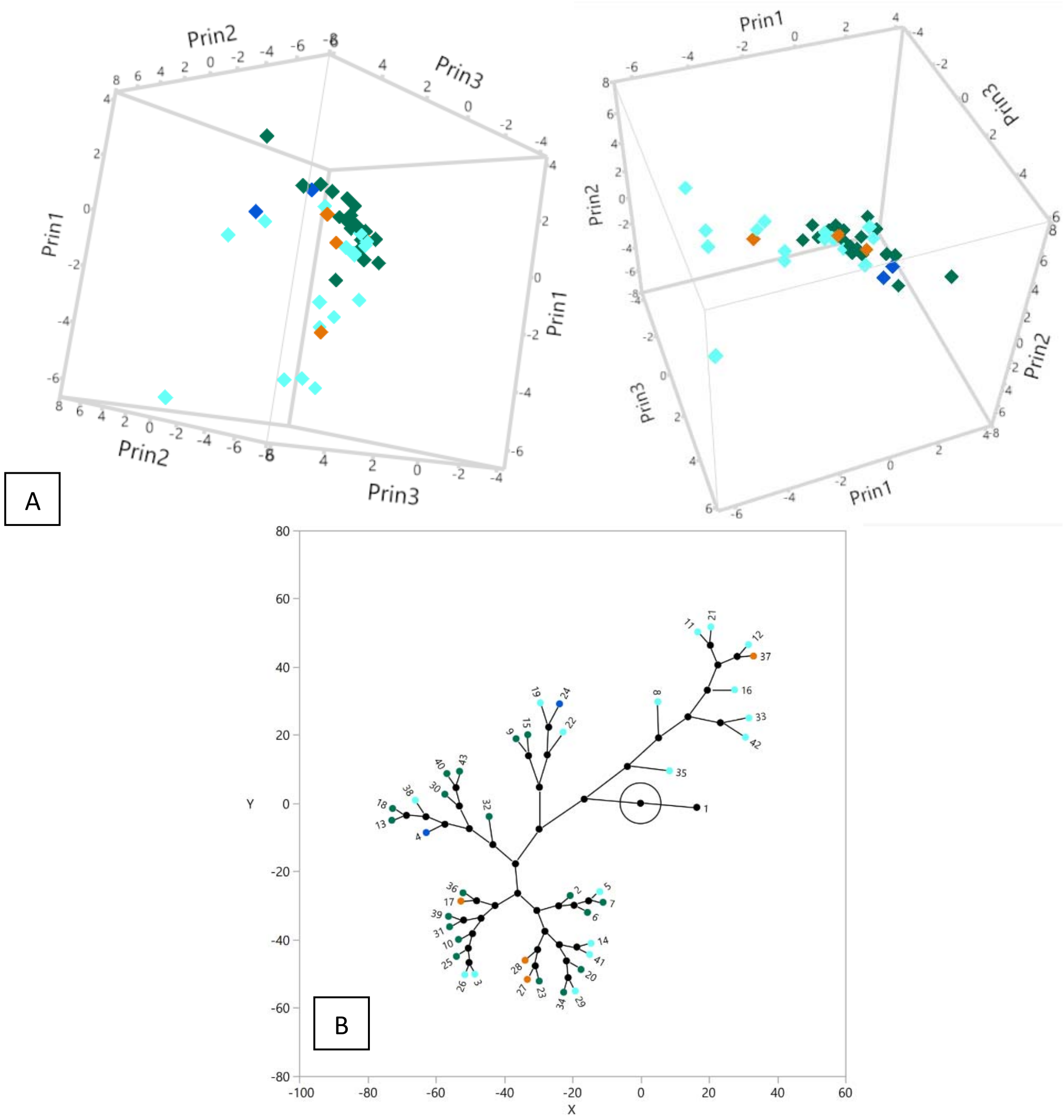

Figure SI 7.2 a) Two rotated views from the same three-dimensional scatter plot of PCA; b) A constellation plot of Hierarchical Cluster analysis using Ward's method. Light blue - spall; Dark blue - fresh wash; Green - casehardening; Orange - casehardening with iron staining. Data from 10 of the 11 study sites: Leang Timpuseng, Leang Lompoa, Gua Jing, Leang Sampeang, Leang Pettakere, Leang Pattae, Leang Bulu Bettue, Leang Barugayya, Leang Sakapao and Leang Jarie.

Leang Balang Pakalu 5 was not ananlysed during initial fieldwork for the pXRF program, but samples from the edge of the panels there were included in the SEM and XRD analyses.

SI SECTION EIGHT.

pXRF Assays.

Key – Spall is blue, case-hardened surfaces orange. Age determinations shown in black, reported after Aubert et al. 2014. Photographs in Figures SI 8.6 to 8.9 taken by Jillian Huntley.

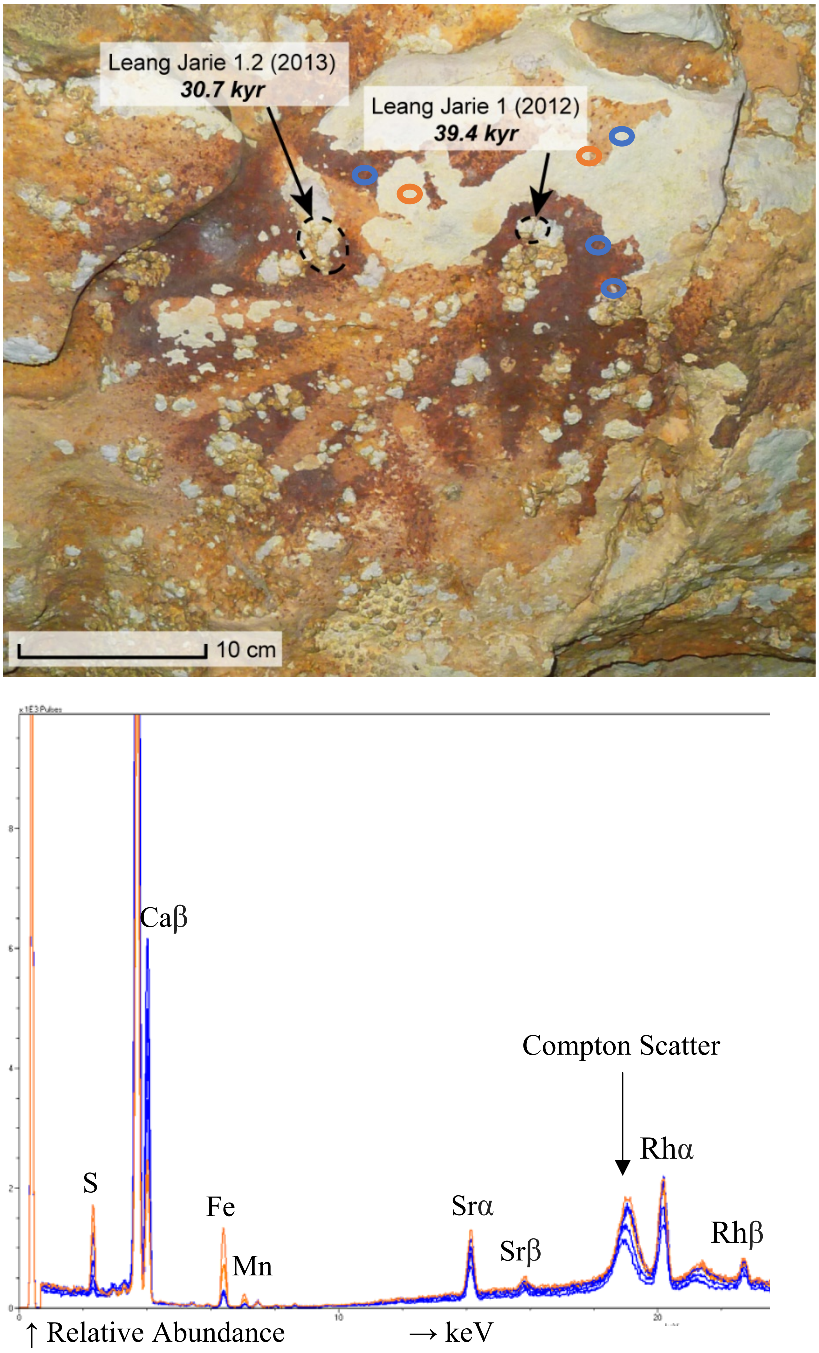

Figure SI 8.1 pXRF spectra collected at Leang Jarie: Top, spectral location relative to previous dating work; Bottom, overly of spectra showing elements diagnostic of haloclasty**.**

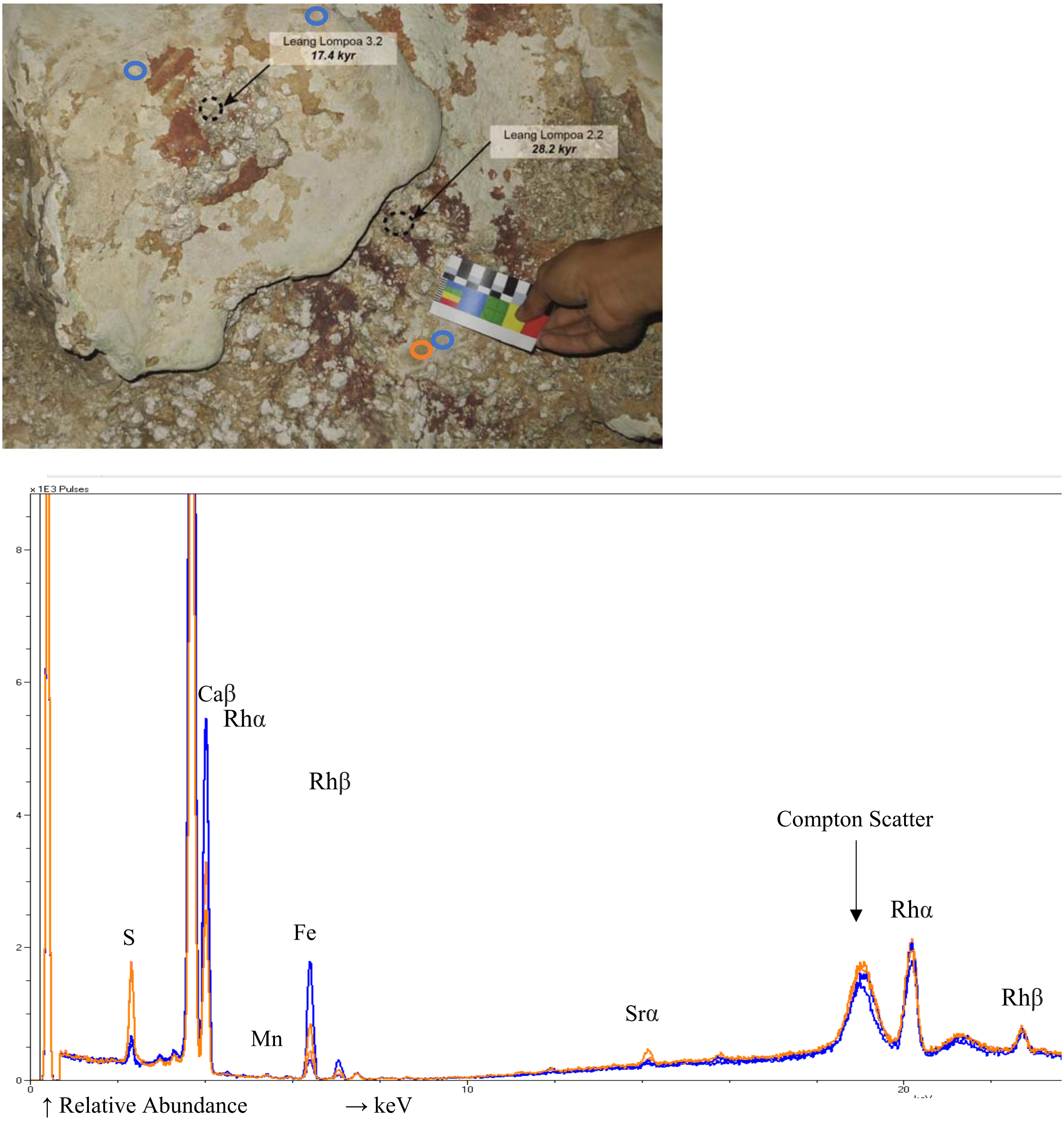

Figure SI 8.2 pXRF spectra collected at Leang Lompoa: Top, spectral location relative to previous dating work; Bottom, overly of spectra showing elements diagnostic of haloclasty**.**

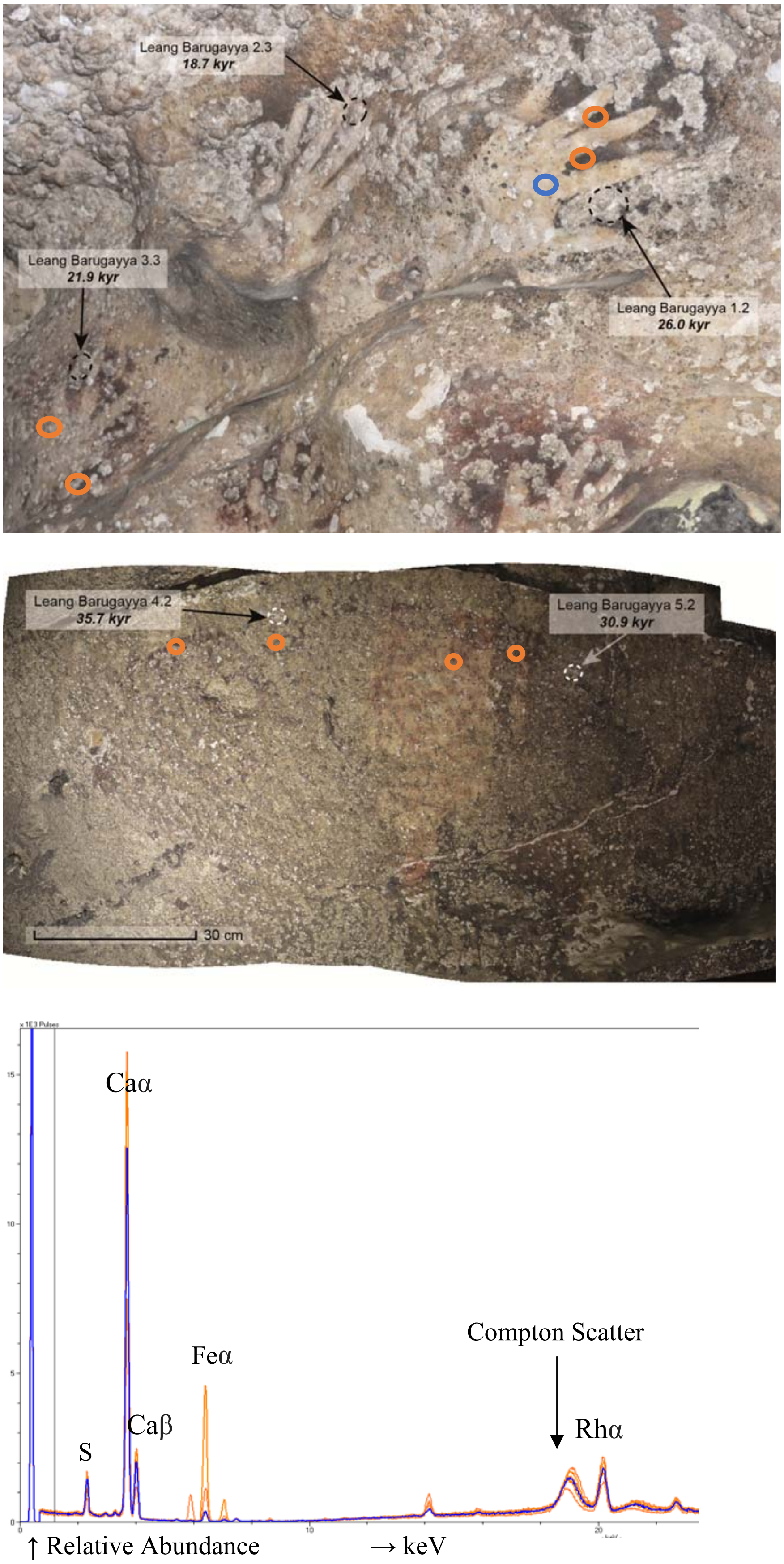

Figure SI 8.3 pXRF spectra collected at Leang Barugayya: Top photographs, spectral locations relative to previous dating work; Bottom, overly of spectra showing elements diagnostic of haloclasty.

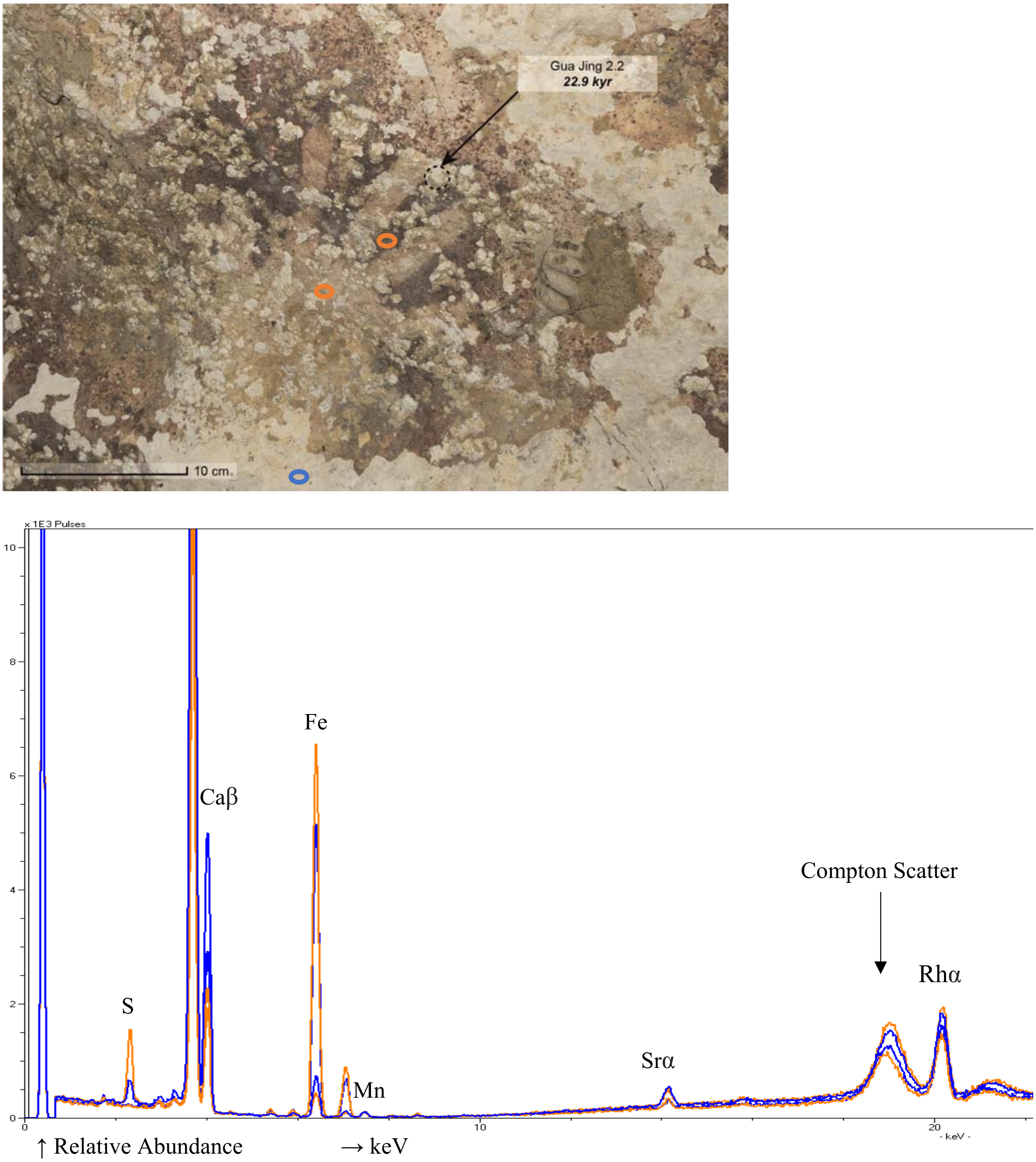

Figure SI 8.4 pXRF spectra collected at Gua Jing: Top, spectral location relative to previous dating work; Bottom, overly of spectra showing elements diagnostic of haloclasty**.**

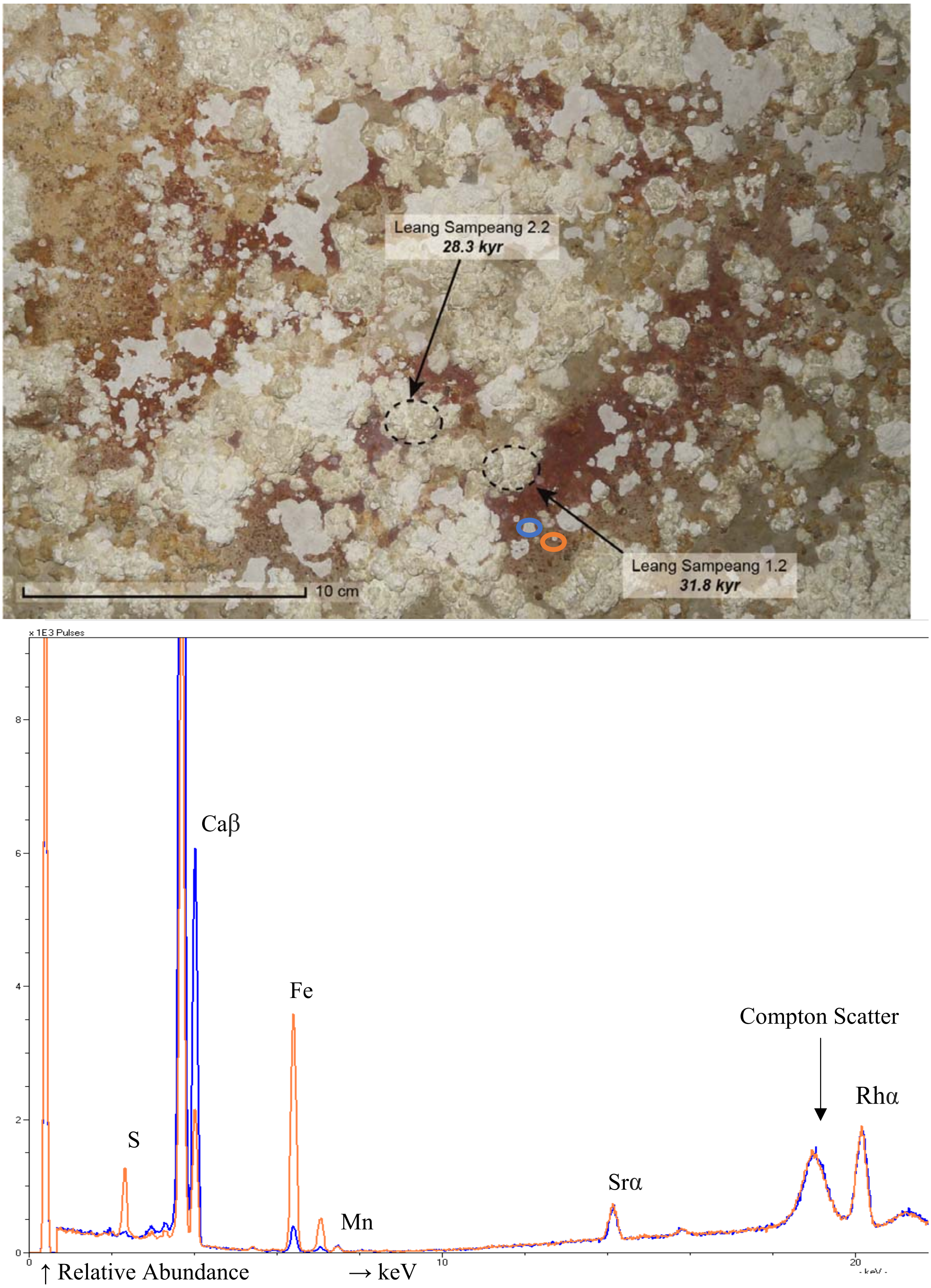

Figure SI 8.5 pXRF spectra collected at Leang Sampeang: Top, spectral location relative to previous dating work; Bottom, overly of spectra showing elements diagnostic of haloclasty**.**
Note: This site is under threat from mining.

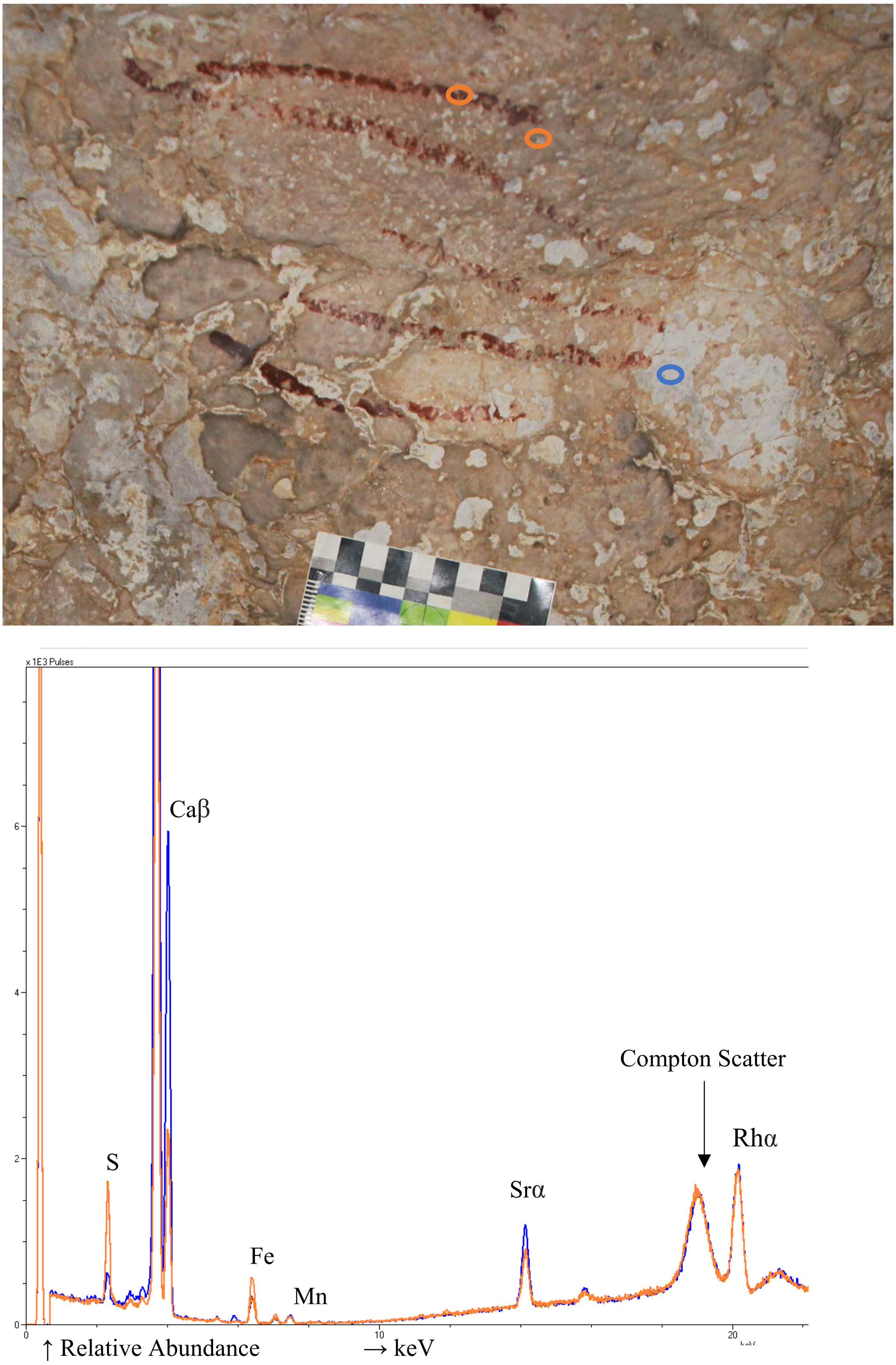

Figure SI 8.6 pXRF spectra collected at Leang Sakapao: Top, spectral location (scale is 10 cm); Bottom, overly of spectra showing elements diagnostic of haloclasty.

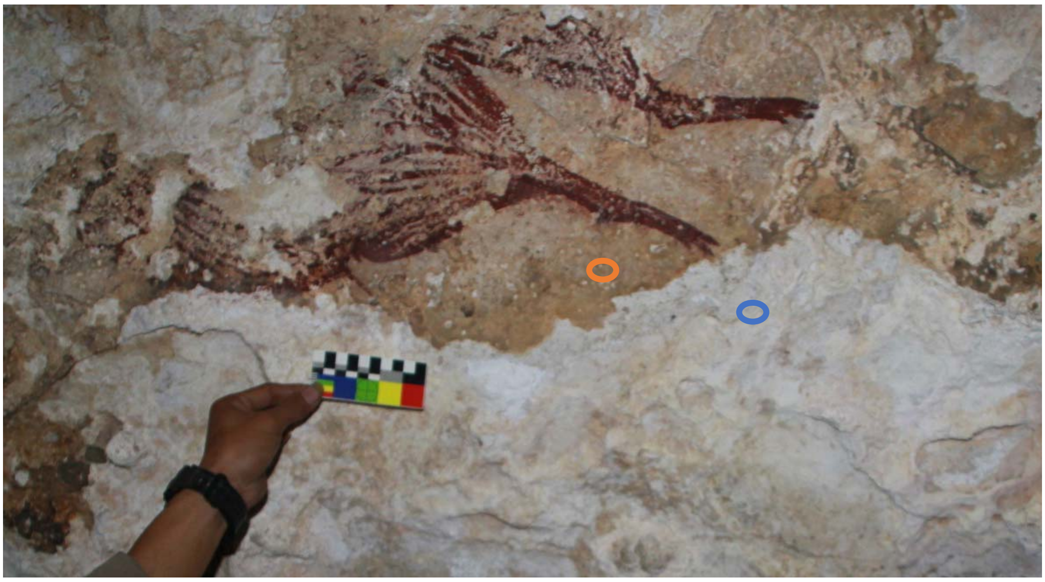

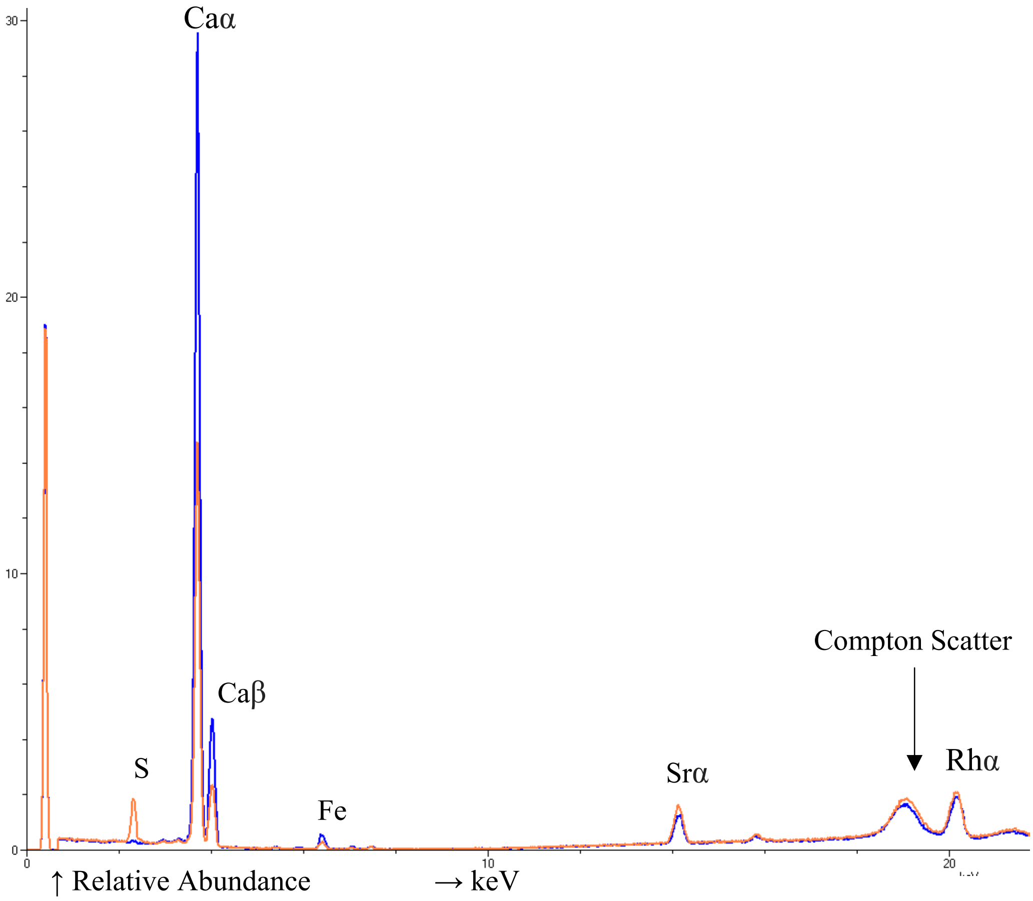

Figure SI 8.7 pXRF spectra collected at Leang Sakapao: Top, spectral location (scale is 10 cm); Bottom, overly of spectra showing elements diagnostic of haloclasty**.**

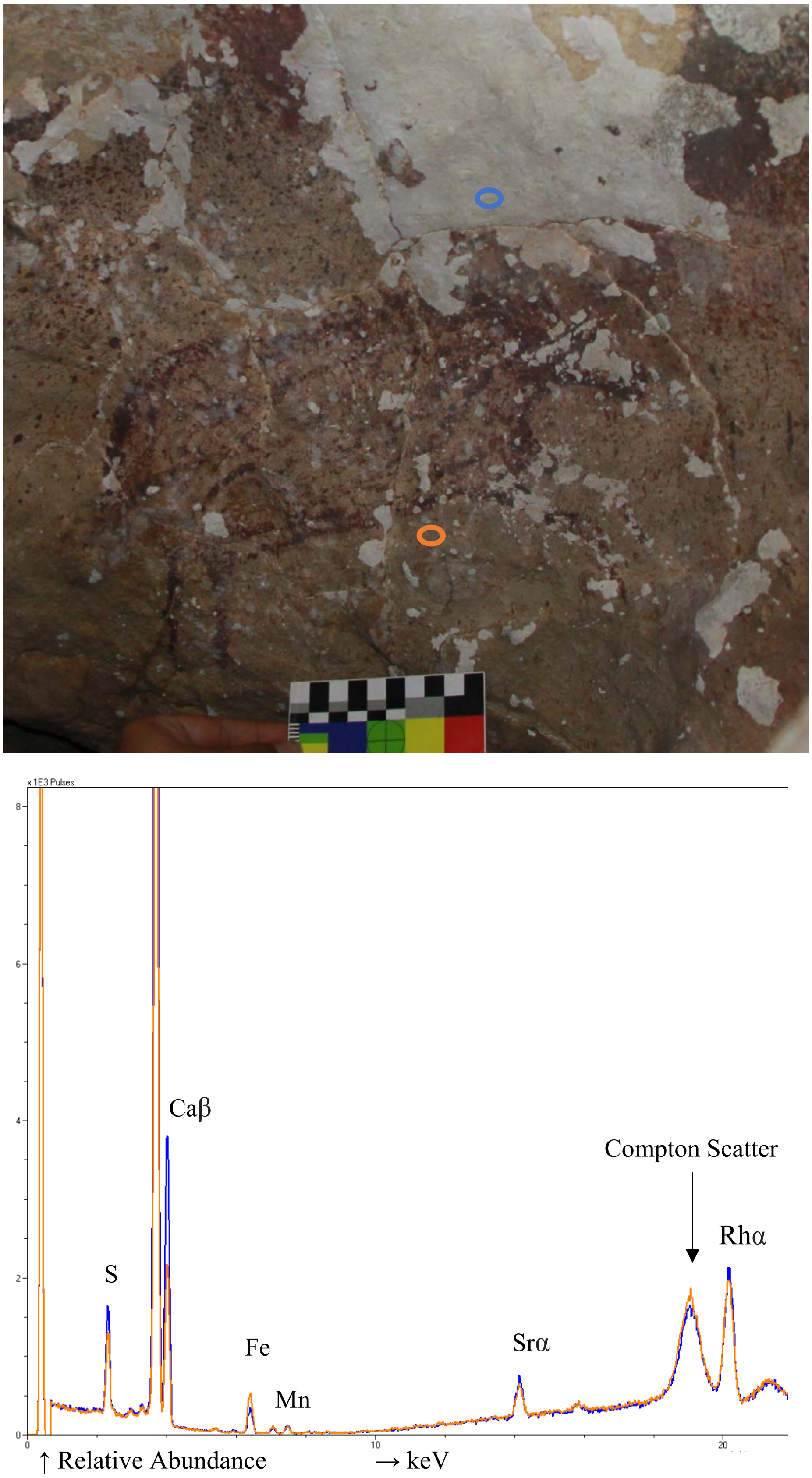

Figure SI 8.8 pXRF spectra collected at Leang Pattae (the same motif illustrated in Figure 3): Top, spectral location (scale is 10 cm); Bottom, overly of spectra showing elements diagnostic of haloclasty.
